# Peptidylarginine deiminase 2 promotes T helper 17-like T cell activation and activated T cell-autonomous death (ACAD) through an endoplasmic reticulum stress and autophagy coupling mechanism

**DOI:** 10.1186/s11658-022-00312-0

**Published:** 2022-03-02

**Authors:** Yi-Fang Yang, Chuang-Ming Wang, I.-Hsin Hsiao, Yi-Liang Liu, Wen-Hao Lin, Chih-Li Lin, Hui-Chih Hung, Guang-Yaw Liu

**Affiliations:** 1 Department of Life Sciences, National Chung Hsing University (NCHU), Taichung, 40227 Taiwan; 2 Ph.D. Program in Tissue Engineering and Regenerative Medicine, National Chung Hsing University, Taichung, 40227 Taiwan; 3 Department of Pediatrics, Ditmanson Medical Foundation Chia-Yi Christian Hospital (CYCH), Chia-Yi, 60002 Taiwan; 4 Institute of Medicine, School of Medicine, Chung Shan Medical University, Taichung, 40201 Taiwan; 5 Department of Allergy, Immunology & Rheumatology, Chung Shan Medical University Hospital, Taichung, 40201 Taiwan; 6 Institute of Genomics and Bioinformatics, National Chung Hsing University (NCHU), Taichung, 40227 Taiwan; 7 iEGG and Animal Biotechnology Center, NCHU, Taichung, 40227 Taiwan

**Keywords:** Peptidylarginine deiminase 2, Cytokines, Activated T cell-autonomous death, Endoplasmic reticulum stress, Autophagy

## Abstract

Peptididylarginine deiminase type 2 (PADI2) catalyzes the conversion of arginine residues to citrulline residues on proteins. We demonstrate that PADI2 induces T cell activation and investigate how PADI2 promotes activated T cell autonomous death (ACAD). In activated Jurkat T cells, overexpression of PADI2 significantly increases citrullinated proteins and induces endoplasmic reticulum (ER) stress and unfolded protein response (UPR) signaling, ultimately resulting in the expression of autophagy-related proteins and autophagy. PADI2 promoted autophagy and resulted in the early degradation of p62 and the light chain 3B (LC3B)-II accumulation. In Jurkat T cells, silencing the autophagy-related gene (Atg) 12 protein inhibits PADI2-mediated autophagy and promotes ER stress and apoptosis, whereas overexpression of Atg12 decreased ER stress and prolonged autophagy to promote cell survival. Additionally, PADI2 regulates T cell activation and the production of Th17 cytokines in Jurkat T cells (interleukins 6, IL-17A, IL-17F, IL-21, and IL-22). In Jurkat T cells, silencing IL-6 promotes autophagy mediated by PADI2 and inhibits PADI2-induced apoptosis, whereas silencing Beclin-1 increases the activation and survival of Th17-like T cells while decreasing autophagy and apoptosis. PADI2 silencing alleviates ER stress caused by PADI2 and decreases cytokine expression associated with Th17-like T cell activation and ACAD. We propose that PADI2 was involved in Th17 lymphocyte ACAD via a mechanism involving ER stress and autophagy that was tightly regulated by PADI2-mediated citrullination. These findings suggest that inhibiting Th17 T cell activation and the development of severe autoimmune diseases may be possible through the use of novel antagonists that specifically target PADI2.

**Supplementary Information:**

The online version contains supplementary material available at 10.1186/s11658-022-00312-0.

## Introduction

Peptidylarginine deiminase (EC 3.5.3.15) is a Ca^2+^-dependent enzyme that converts arginine residues in proteins to citrulline residues [1]. PADI is classified into five isotypes (1–4 and 6), each with its own unique substrate specificity and tissue expression pattern [1]. PADI2 is expressed in a variety of tissues, including the brain, skeletal muscle, spinal cord, oligodendrocytes, uterus, pancreas, salivary gland, pituitary gland, sweat gland, spleen, macrophages, bone marrow, and leukocytes [2]. PADI-mediated posttranslational modification (deimination or citrullination) decreases the net charge of protein substrates, resulting in the loss of potential ionic bonds or rearrangement of hydrogen bonds, which ultimately results in protein unfolding [3].

Unfolded protein aggregates and damaged organelles are removed from cells by autophagy, a major intracellular catabolic mechanism [4]. Autophagy can keep cells alive by recycling nutrients, maintaining cellular energy homeostasis, and degrading toxic cytoplasmic constituents. Autophagy is the process by which autophagosomes mature and degrade. Autophagosomes fuse with lysosomes to form autophagolysosomes and degrade their cargo. Excessive autophagy in response to cellular stress may cause cell death. Autophagy is involved in development, differentiation, stress, infection, neurodegenerative diseases, and cancer. During autophagy, cytoplasmic cargo is transported to the lysosome via vesicles. Autophagy-related genes (Atgs), such as Atg5, Atg7, and Atg12 are necessary for vesicle elongation and autophagosome completion. Atg7 plays a critical role in autophagosome regulation, and Atg5 and Atg12 can form intracellular complexes required for autophagosome elongation. These complexes are activated by Atg7 and are required for the light chain 3B (LC3B) ubiquitin-like conjugation system to function properly [5].

LC3B and sequestosome 1 (SQSTM1) are the two primary autophagy markers. SQSTM1, referred to in humans as p62, is a ubiquitin-binding scaffold protein that marks proteins for degradation via autophagy. When autophagy is inhibited, p62 accumulates within cells; however, when autophagy is induced, protein levels decrease. The LC3B marker exists in two forms: unconjugated LC3B-I (18 kDa) found in the cytosol, and phosphatidylethanolamine-conjugated LC3B-II (16 kDa) found in the autophagosomal membrane [6]. Beclin-1 is the human homolog of yeast Atg6, which is required for the formation of the autophagosome membrane in collaboration with the mammalian class III phosphoinositide 3-kinase (PI3K). Beclin-1 interacts with Bcl-2 and acts as a tumor suppressor, thereby establishing a link between autophagy and tumorigenesis [7]. Members of the Bcl-2 family can inhibit the interaction between Beclin-1 and PI3K [8]. Several members of the anti-apoptotic Bcl-2 family, including Bcl-2, Bcl-xL, and Mcl-1, contain four Bcl-2 homology domains and inhibit autophagy by interacting with Beclin-1. T lymphocytes are susceptible to both proapoptotic and antiapoptotic effectors, such as members of the Bcl-2 family. Indeed, several molecules, including Beclin-1, caspase-9, and PI3K class I, are involved in both autophagy and apoptosis regulation [9]. Thus, a double-edged sword has been proposed for autophagy, as autophagy can act as a protector or a killer, depending on the surrounding cellular environment [10]. Additionally, it has been established that dysregulated autophagy plays a significant role in a variety of human diseases, including inflammatory disorders [11], neurodegeneration [12, 13], intracellular pathogen infections [4], and cancer [14].

Lysosome-associated membrane protein-1 (LAMP-1) and lysosome-associated membrane protein-2 (LAMP-2) are two major lysosomal membrane proteins [15]. LAMP-1 and LAMP-2 are required for lysosome-mediated regulation of autophagosome–lysosome fusion rates, and lysosomes control autophagic flux by acting at both the initiation and termination phases [16].

The endoplasmic reticulum (ER) is a eukaryotic cellular component that plays a critical role in the synthesis and secretion of membrane proteins, lipid synthesis, and glucose metabolism [17]. ER stress occurs when the luminal ER homeostasis is disrupted, as a result of reactive oxygen species (ROS) generation, hypoxia, protein unfolding, and changed energy levels and Ca^2+^ concentrations. Unfolded proteins in the endoplasmic reticulum (ER) initiate an adaptive ER stress response dubbed the unfolded protein response (UPR). The UPR pathway is essential for developmental processes requiring protein synthesis and export, such as differentiating T lymphocyte into cytokine-secreting killer cells [18, 19]. Additionally, the UPR is required for the differentiation and growth of mature antigen-presenting cells.

The UPR can be triggered through ER membrane receptors such as protein kinase RNA-like ER kinase (PERK), inositol-requiring enzyme 1 (IRE1α), and activating transcription factor 6 (ATF6); activating transcription factors 4 (ATF4) is also involved in PERK-eIF2α signaling [20]. UPR has been shown to enhance glucose-regulated protein 78 (GRP78/Bip) dissociation from these receptors. GRP78 is required for the proper folding of proteins and the prevention of protein aggregation in the ER lumen [21]. In addition, the UPR activates PERK. Additionally, transcripts encoding C/EBP homologous protein (CHOP) and X-box binding protein 1 (XBP1) are indicators for ER stress. CHOP is a transcription factor (TF) associated with ER stress-induced apoptosis [22], where it induces apoptosis through posttranslational stabilization of Bim, a proapoptotic member of the Bcl-2 family [23]. By controlling the ER membrane kinase/nuclease IRE1α, Bcl-2-associated X protein (Bax) and BCL2-antagonist/killer 1 (Bak) can modulate ER stress. IRE1α is a nuclease that is involved in the alternative splicing of XBP1 from unspliced XBP1 (uXBP1) to spliced XBP1 (sXBP1), which is a critical modulator of the UPR. XBP1 has been shown to activate autophagy genes such as Atg5, Atg7, and Atg8 [24]. Additionally, the UPR is involved in the mitochondrial apoptotic pathway via its control of the Bcl-2 family. On the ER membrane, the Bcl-2 family suppresses pro-apoptotic proteins such as Bax/Bak and BH3-only proteins [25].

Apoptosis is the signaling pathway that leads to programmed cell death (PCD). Apoptosis has been researched in many disease conditions, such as rheumatoid arthritis (RA), Parkinson’s, and Alzheimer’s diseases. Apoptosis is triggered by DNA damage, which triggers the mitochondrial pathway (intrinsic pathway) [26]. When external insults trigger apoptosis, a particular endonuclease degrades DNA, resulting in oligonucleosomal-sized fragments [27]. Caspases are a large protein family involved in apoptosis (caspase-3, caspase-4, caspase-6, caspase-8, and caspase-9) and inflammation (caspase-1). Caspase-3 is triggered by proteolytic processing and then cleaves downstream targets of Poly (ADP-ribose) polymerase (PARP), irreversibly killing the cell.

The process of apoptosis in activated T cells mediated by the Bcl-2 family is referred to as activated T cell-autonomous death (ACAD) [28] and is responsible for the death of the majority of T cells responding to foreign antigens. The Bcl-2 family Bax and Bak (pro-apoptotic) proteins can promote mitochondrial permeability, while Bcl-2 and Bcl-xL (anti-apoptotic) proteins can block apoptotic proteins. Caspase-3 activates PARP cleavage, causing nuclear damage and apoptosis. T helper cells are a kind of T cell vital to the adaptive immune system. These cells can either stimulate or suppress immune responses based on their cytokines. Effector T helper cells can create cytokines or proteins to activate other leukocytes. Memory T helper cells retain antigen affinity and provide rapid immune protection against recognized pathogens. Regulatory T cells (Tregs) regulate a number of immune responses to maintain immune homeostasis. Th17 cells generate interleukin-17 (IL-17) and are activated by interleukin-6 (IL-6) and transforming growth factor β (TGFβ). Th17 cells produce IL-17A, IL-21, and IL-22, as well as the transcription factors STAT3 and RORt. Interferon gamma (IFNγ) and interleukin-4 (IL-4) have been shown to inhibit Th17 development. Th17 cells can reprogramme to become protective or proinflammatory pathogenic cells. IL-6 modulates the complement system, killing extracellular bacteria and fungi. Complement-mediated hypersensitivity occurs when Th17 cells over-activate autoantigens. Psoriasis, inflammatory bowel disease, RA, and Crohn’s disease all have activated Th17 cells [29, 30]. Th17 also has a function in allergic airway inflammation [31]. IL-21 and IL-22 are secreted by Th17 cells and have been shown to enhance Th17 numbers [32].

Autoimmunity is mediated also by PADIs and citrullinated proteins. PADI2 is found critical in the pathogenesis of neurodegenerative diseases in humans, such as multiple sclerosis (MS) and Alzheimer’s disease (AD) [33]. We previously demonstrated that PADI2 has an effect on signaling cascades involved in cell survival, proliferation, and stress response [34]. The goal of this work was to establish whether PADI2 promotes T helper cell activation and ACAD in T lymphocytes and to characterize the T helper cell subtypes activated by PADI2-mediated protein citrullination. Additionally, we attempted to unravel the tightly controlled pathways behind PADI2 activation, ER stress, and activation of the lysosome and autophagy during ACAD.

## Materials and methods

### Cell culture and treatment

The Jurkat T cells were obtained from Bioresource Collection and Research Center (BCRC, Hsinchu, Taiwan), DND-41, and P12-ICHIKAWA cells were purchased from DSMZ-German Collection of Microorganisms and Cell Cultures GmbH. Jurkat T, DND-41, and P12-ICHIKAWA cells were cultured in 90% RPMI 1640 medium (Gibco BRL, Thermo Fisher Scientific, Waltham, MA, USA) with 10% FBS at 37 °C in a humidified incubator containing 5% CO_2_. Acridine orange (AO), doxycycline (Dox), 12-O-tetradecanoylphorbol-13-acetate (TPA), and ionomycin (Ion) were purchased from Sigma Aldrich (St. Louis, MO, USA).

### Cell viability and acridine orange (AO) staining assay

A trypan blue assay was used to count the cells. The viability of cells was determined by dividing the number of viable cells in the experimental groups by the number of viable cells in the control group. On each slide, 1 × 10^6^ cells were stained with 1 μg/mL Acridine orange (AO) solution (Sigma Aldrich, St. Louis, MO, USA) for 5 min at room temperature, after that, cells were washed with phosphate-buffered saline (PBS) and re-suspended with 10 µL of PBS, then a fluorescence microscope (Olympus America, Center Valley, PA, USA) was used to detect green fluorescence and red fluorescence. Autophagic vacuoles (AVOs) and apoptotic cell death were measured by dividing the number of fluorescent autophagosome and fluorescent nuclei (apoptotic cells) by the total number of cells counted in six randomly chosen high-power fields.

### Cell transfection

Human PADI2 cDNA was purchased from RZPD-German Science Centre for Genome Research. A sense primer (5′-GCGGCCGCATGCTGCGCGA-3′) having a 5′ NotI site and an antisense primer (5′-GTCGACCACCAAAAGA-3′) including a 5′ SalI site were used to amplify PADI2 cDNA. The polymerase chain reaction (PCR) product was ligated into a T vector and transformed into *E. coli* strain JM109. Overnight at 37 °C, the bacteria were cultivated, the plasmids were eluted, and the amplified plasmids were digested with NotI–SalI and subcloned into the NotI–EcoRI site of the pTRE2hyg Tet-On vector (BD Biosciences Clontech, Palo Alto, CA, USA). Using calcium phosphate-mediated transfection, the pTRE2hyg-PADI2 and pTRE2hyg Tet-On system vectors were stably transfected into Jurkat T cells. Hygromycin (400 μg/mL) was used to select stable transfected cells. The Jurkat Tet-On cell system was triggered by adding Dox to the growth media.

### Protein expression knockdown by shRNA technology

The PADI2, Atg5, beclin-1 (BECN1), IL-6, and Atg12 shRNA gene silencers were obtained from National RNAi Core Facility (Academia Sinica, Taipei, Taiwan). Stable knockdown of Atg5, BECN1, and Atg12 in the Tet-On-PADI2 Jurkat T cells were transfected with calcium phosphate (0.8 µg/mL) and selected with puromycin (2 µg/mL). The control cells stably expressed shLuc (pLKO).

### Protein overexpression of *atg12* gene

The human Atg12 gene was purchased from Addgene (Cambridge, MA, USA). Polymerase chain reaction (PCR) amplification of the encoding region of human atg12 cDNA was performed with our designed primers derived from the human atg12 sequences. The plasmids encoding Atg12 were generated by adding the 564 bps EcoRI–HindIII coding region fragment. The PCR products were sequenced and sub-cloned to eukaryotic expression vector, pCMV-Tag (Novagen, Madison, WI, USA).

### Colorimetric assay for PAD2 enzyme activity (for cell-based assay)

The enzymatic activity of PAD2 in cells was determined using a colorimetric method based on the rate of formation of peptidylcitrulline [35, 36]. In general, an assay solution containing 100 mM Tris–HCl (pH 7.5), 10 mM CaCl_2_, 2.5 mM DTT, and 10 mM benzoyl-l-arginine ethyl ester (the artificial substrate for PAD2) was incubated at 37 °C for 1 h with cell lysates (1.5–2 mg/sample). The reaction was terminated by adding 12.5 μL of 5 M perchloric acid and centrifuging the pellet to remove it. The soluble fraction was treated with 150 μL of carbidino detection reagent containing one part 0.5% diacetyl monoxime and 0.01% thiosemicarbazide and two parts 0.25 mg/mL FeCl_3_ in a solution containing 24.5% sulfuric acid (H_2_SO_4_) and 17% phosphoric acid (H_3_PO_4_). The mixture above was then heated to 110 °C for 5 min. After heating, the absorbance of the sample was measured at 535 nm was determined using a PARADIGM Detection Platform microplate reader (Beckman Coulter, USA). To construct a standard curve, citrulline concentrations ranging from 0 to 40 nmol were determined.

### Luciferase reporter assay

Transient transfections of Tet-On-PADI2 cells with calcium phosphate were conducted using 15 µg of the IL-4 promoter [37], IL-2 promoter, IFNγ promoter, IL-17 promoter, pTATA-tk-Luc (containing a STAT3-binding site), and 5 µg of pCMV-β-gal plasmids at 37 °C for 12 h (Addgene, MA, USA). Following 12 h of Dox treatment, the medium was changed and the cells extracted for a reporter assay. Luciferase experiments were carried out using a Promega dual-luciferase reporter assay kit (Promega Corporation, Madison, WI, USA), and activity was determined using a TR 717™ Microplate Luminometer (Thermo Fisher Scientific, Waltham, MA, USA). As an internal check for transfection efficiency, β-galactosidase activity was determined by staining with the chromogenic substrate ortho-nitrophenyl-β-galactoside.

### Detection of intracellular ROS

The level of intracellular ROS was determined by flow cytometry using the oxidation-sensitive fluorescent dye 2′-7′-dichlorodihydrofluorescein diacetate (DCFH-DA). Jurkat T cells were planted in 60-mm cell culture dishes and treated for 12 h at 37 °C and 5% CO_2_ with or without Dox. After thorough washes with PBS and suspension in PBS, the cells were incubated at 37 °C for 25 min with DCFH-DA. Flow cytometry was then used to detect the DCF fluorescence distribution using 10,000 cells at an excitation wavelength of 488 nm and an emission wavelength of 530 nm.

### DNA fragmentation analysis

Cells (5 × 10^6^) were harvested and lysed in a digestion buffer (0.5% sarkosyl, 0.5 mg/mL proteinase K, 50 mM Tris–HCl, pH 8.0, and 10 mM EDTA) at 55 °C overnight. The cells were subsequently treated with 0.5 μg/mL RNase A for 2 h. The genomic DNA was extracted using phenol–chloroform–isoamyl alcohol. The reaction products were analyzed by gel electrophoresis at 50 V for 25 min with 2% agarose. Approximately 20 μg of genomic DNA was loaded in each well; the resulting bands were visualized under ultraviolet (UV) light and photographed.

### Western blotting analysis

The cells (1 × 10^6^ cells/mL) were harvested overnight at 37 °C and 5% CO_2_. To purify the total proteins, the cells were lysed in cold lysis buffer (10% v/v glycerol, 1% v/v Triton X-100, 1 mM sodium orthovanadate, 1 mM EGTA, 10 mM NaF, 1 mM sodium pyrophosphate, 20 mM Tris, pH 7.9, 100 μM β-glycerophosphate, 137 mM NaCl, 5 mM EDTA, 1 mM PMSF, 10 μg/mL aprotinin, and 10 μg/mL leupeptin) and subsequently homogenized and centrifuged. The supernatants were boiled in a loading buffer, and an aliquot corresponding to 30–50 μg of protein was separated by sodium dodecyl sulfate–polyacrylamide gel electrophoresis (SDS-PAGE). The proteins were probed with relevant antibodies (1:1000 dilution) for 3 h at room temperature or overnight at 4 °C. Then, the proteins were incubated with a secondary antibody conjugated with horseradish peroxidase for 1 h at room temperature. The immune-reactive proteins were visualized by enhanced chemiluminescence.

The antibodies used in immunoblot includes anti-PADI2 (MDBio, Taipei, Taiwan), anti-citrulline antibodies (#07-377, Upstate Biotechnology, Lake Placid, NY, USA), anti-apoptotic protein antibodies anti-Cyt-c, anti-Apaf1, anti-caspase-3, anti-caspase-9, anti-PARP, anti-Bcl-xL and anti-Bax (Cell Signaling Technology, Inc., Danvers, MA, USA), antibodies against mTOR, p-mTOR, AKT, and p-AKT (Cell Signaling Technology, Inc., Danvers, MA, USA), antibodies against ER stress-related protein PERK, p-PERK, GRP78, IRE1, p-eIF2α and CHOP (Cell Signaling Technology, Inc., Danvers, MA, USA), antibodies against autophagic protein Atg12-Atg5, Atg12, Atg5, Atg7, p62 and Beclin-1 (Cell Signaling Technology, Inc., Danvers, MA, USA), antibodies against LC3B (#2775, Cell Signaling Technology, Inc., Danvers, MA, USA), antibodies against the lysosomal protein LAMP-1 and LAMP-2 (Cell Signaling Technology, Inc., Danvers, MA, USA), and antibodies against β-actin (Santa Cruz Biotechnology, Inc., Dallas, TX, USA).

### Analysis of mRNA expression by reverse transcription (RT)-PCR analysis

Total RNA was isolated from 1 × 10^6^ cells with TRIzol reagent (Thermo Fisher Scientific Inc., Waltham, MA, USA). Reverse transcription was performed using M-MLV Reverse Transcriptase (Promega Corporation, Madison, WI, USA). A total of 1 µL of cDNA was used for PCR amplification over 25–36 cycles with denaturation at 95 °C for 30 s, annealing at 60 °C for 90 s and extension at 72 °C for 60 s. The reaction products were semi-quantified by electrophoresis using a 2% agarose gel. The primer sequences used to amplify the target genes were shown in Additional file 1: Table S1.

### Statistical analyses

The data shown represent the mean ± standard error of mean (SEM). Statistical analysis was performed by one-way or two-way analysis of variance (ANOVA) at significance levels of *p* < 0.05 (*), *p* < 0.01 (**), and *p* < 0.001 (***).

## Results and discussion

### Activation of T-cell acute lymphoid leukemia (T-ALL) cells results in the expression of PADI2, as well as autophagy and apoptosis

**Fig. 1:**
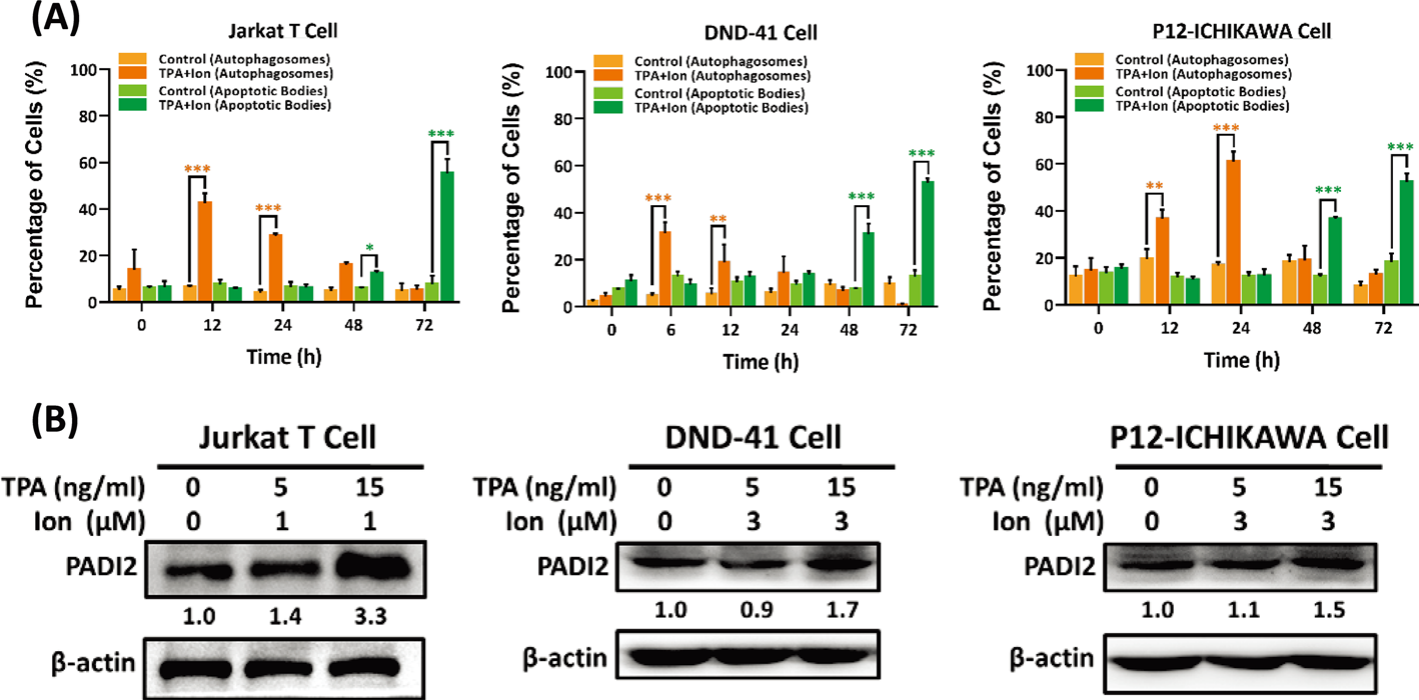
Induction of autophagy and apoptosis in T-ALL cell lines, Jurkat T, DND-41, and P12-ICHIKAWA by TPA and ionomycin (Ion). **A** The bar graphs derived from acridine orange (AO) staining images of Jurkat T, DND-41, and P12-ICHIKAWA cells (Additional file 1: Fig. S1A) show the percentages of the cell with autophagosomes or apoptotic bodies. The data are presented as the mean ± SEM of three separate experiments (*p < 0.05, **p < 0.01, and ***p < 0.001). The *p* value in was calculated by one-way ANOVA. **B** Proteins extracted from Jurkat T, DND-41, and P12-ICHIKAWA cells were detected using antibodies against PADI2 and β-actin. The value indicates the ratio of protein signal to β-actin signal.

Numerous studies have demonstrated a high level of citrullinated proteins in activated T cells during the autoimmune response [38]. Citrullinated proteins are the result of peptides being modified posttranslationally by PADIs. The purpose of this study was to determine whether PADI2 is involved in the activation of Jurkat T cells and in the development of ACAD. The T-cell acute lymphoid leukemia (T-ALL) cell lines Jurkat T, DND-41, and P12-ICHIKAWA are activated using 12-O-tetradecanoylphorbol-13-acetate (TPA) and ionomycin (Ion). Acridine orange (AO) staining revealed acidic vesicular organelles with autophagic vacuoles (AVs) and apoptotic bodies in all three cell lines (Additional file 1: Fig. S1A); The percentage of cells with autophagosomes and apoptotic bodies was plotted against time in Fig. 1A, indicating that autophagy (6–24 h) occurred earlier than apoptosis (24–72 h). PADI2 protein expression was also significantly increased in TPA/Ion activated T-ALL cells (Fig. 1B); The cell viability of the three activated T-ALL cells also decreased in a dose and time-dependent manner (Additional file 1: Fig. S1B). These findings indicate that activating T-ALL cells causes PADI2 expression, as well as early autophagy and apoptosis.

### PADI2 overexpression exacerbates apoptosis in activated Jurkat T cells

**Fig. 2:**
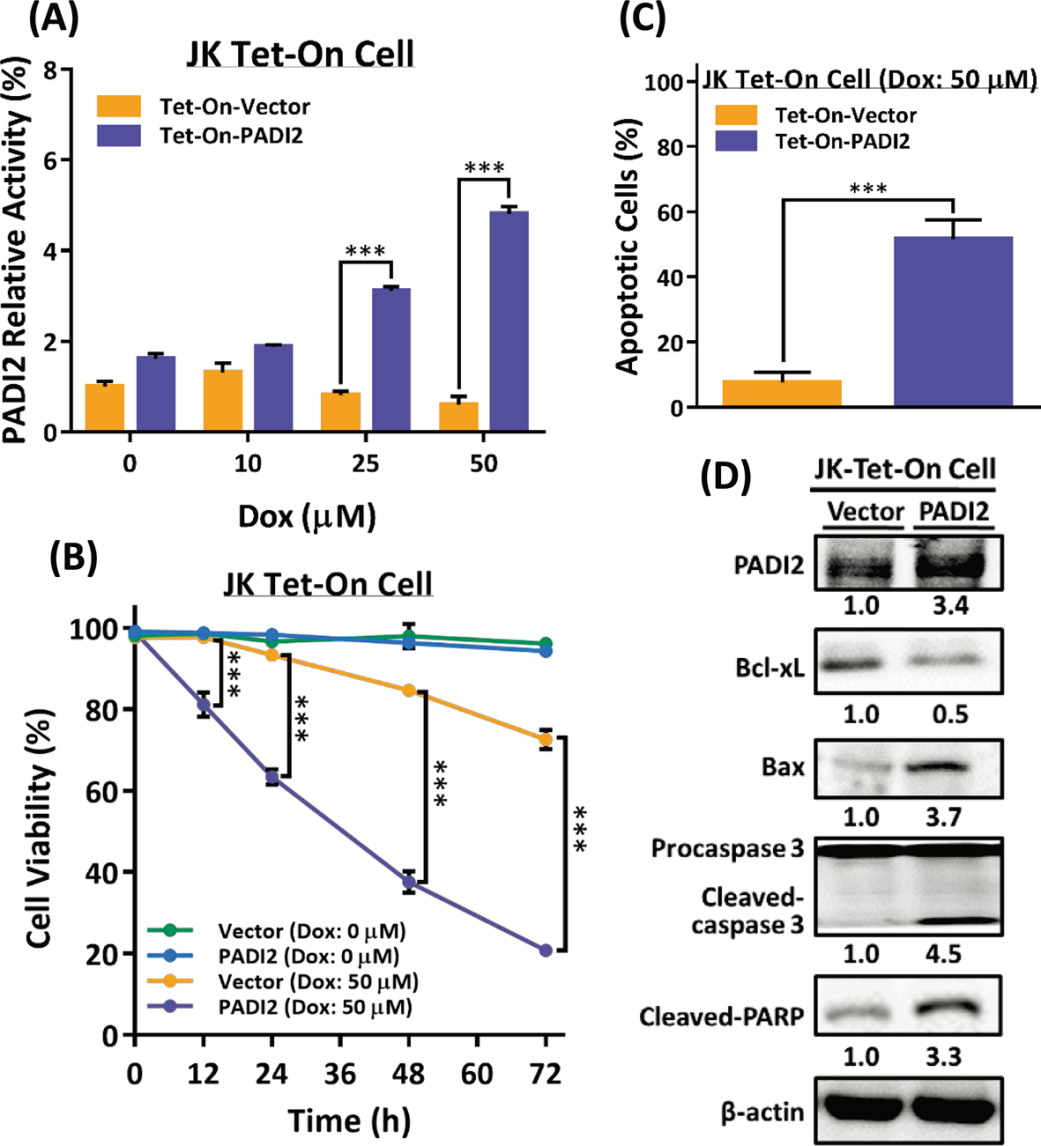
PADI2 overexpression increases apoptosis in Jurkat T cells. **A** The colorimetric method was used to determine the activity of the PADI2 enzyme [60]. The data are presented as the mean ± SEM of three independent experiments (***p < 0.001). The *p* value in was determined by one-way ANOVA. **B** Cell viability of Tet-On-Vector and Tet-On-PADI2 cells without or with 50 μM Dox treatment was determined by trypan blue exclusion. The data are presented as the mean ± SEM of three independent experiments (***p < 0.001). The *p* value in was determined by one-way ANOVA. **C** The bar graphs show the percentages of cells with apoptotic bodies with 50 μM Dox treatment for 48 h. The data are presented as the mean ± SEM of three separate experiments (***p < 0.001). The *p* value in was calculated by Student’s t-test. **D** Antibodies against PADI2, Bcl-xL, Bax, cleaved-caspase 3, cleaved-PARP, and β-actin were used to detect proteins extracted from Tet-On-Vector and Tet-On-PADI2 cells 48 h after exposure to 50 μM Dox. The value indicates the ratio of protein signal to β-actin signal

PADI2 proteins were induced in activated Jurkat T cells, and autophagy markers LC3B and apoptotic markers cleaved-caspase-3 proteins were also significantly increased time-dependently (Additional file 1: Fig. S2A). To examine the effect of PADI2 on autophagy and apoptosis in the cell, we used a transcriptional regulation system (Tet-on) to overexpress PAD2 in Jurkat T cells under the inducible control of doxycycline (Dox) [35, 36]. Tet-On-PADI2 cells expressed significantly more PADI2 protein and mRNA than Tet-On-Vector cells upon Dox induction (Additional file 1: Fig. S2B, C, respectively); additionally, Tet-On-PADI2 cells expressed three to fivefold the activity of the PADI2 enzyme compared to Tet-On-Vector cells (Fig. 2A).

Our current findings unequivocally demonstrate that overexpression of PADI2 decreases cell viability while simultaneously inducing apoptosis (Fig. 2). After 48 h of Dox treatment, light microscopy revealed that Tet-On-PADI2 cells developed round and lobulated shapes (Additional file 1: Fig. S2D); the viability of Tet-On-PADI2 cells was significantly decreased over time (Fig. 2B), DNA gel electrophoresis revealed that DNA fragmentation only observed in Tet-On-PADI2 cells (Additional file 1: Fig. S2E), and the percentage of cells with apoptotic bodies was significantly higher in Tet-On-PADI2 cells than in Tet-On-Vector cells (Fig. 2C). Meanwhile, overexpression and activation of PADI2 drastically lowered antiapoptotic protein Bcl-xL expression and considerably increased pro-apoptotic protein Bax, as well as cleaved caspase-3 and cleaved-PARP expression after Dox treatment for 48 h (Fig. 2D). These morphological and molecular alterations are consistent with ACAD. PADI2 induced autophagy and apoptosis, resulting in Jurkat T cells undergoing ACAD.

### Overexpression of PADI2 increases citrullinated proteins and induces autophagy-related proteins in activated Jurkat T cells

**Fig. 3:**
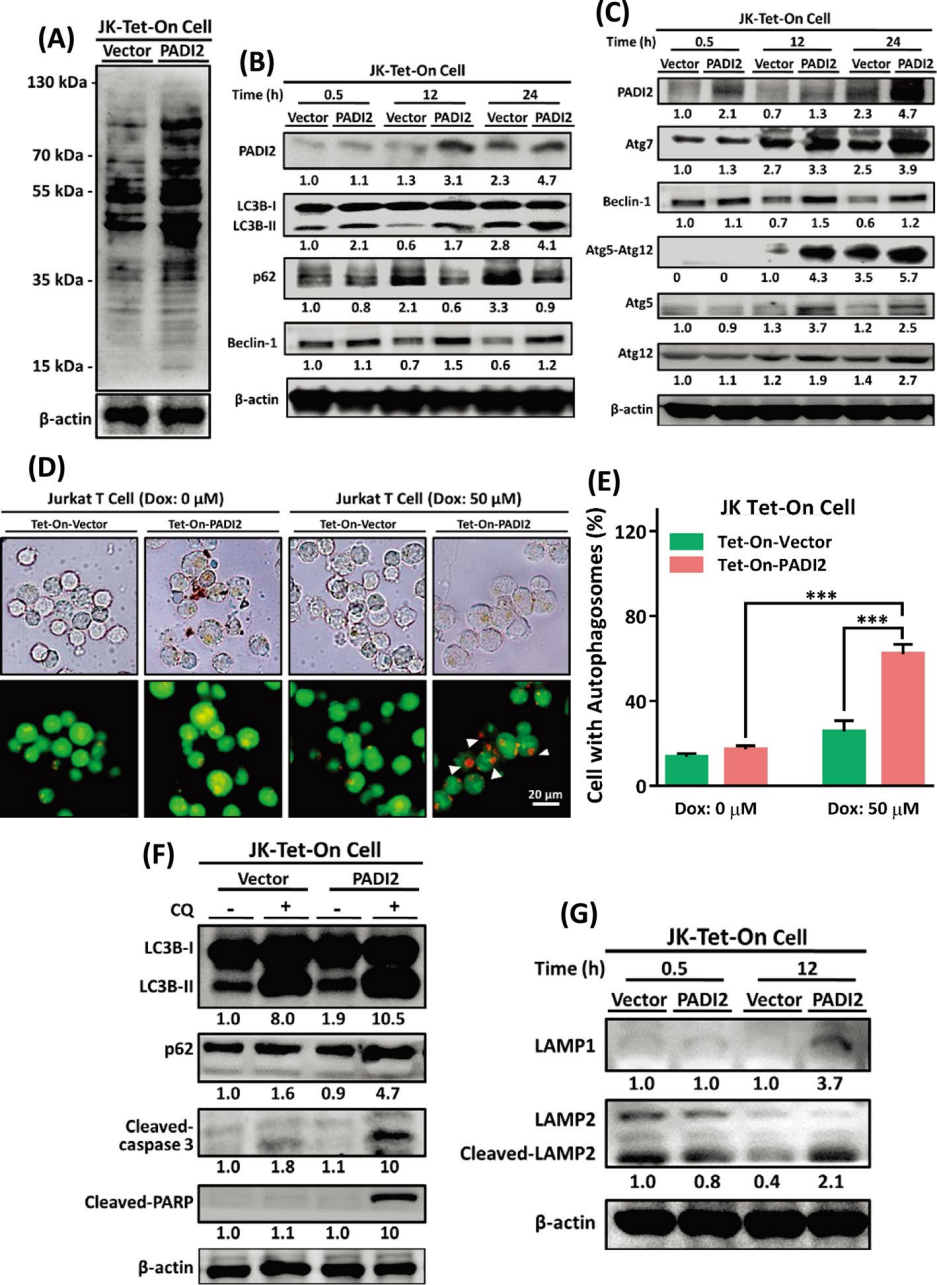
Overexpressing PADI2 increases citrullinated proteins and autophagy-related proteins expression in activated Jurkat T cells. **A** Jurkat Tet-On-Vector and Tet-On-PADI2 cells were treated with 50 μM Dox for 12 h. Anti-citrulline antibodies were used to immunoblot lysates from cells. **B** Proteins extracted from Tet-On-Vector and Tet-On-PADI2 cells were detected using antibodies against PADI2, p62, LC3B, and β-actin as indicated time after 50 μM Dox exposure. **C** Proteins extracted from Tet-On-Vector and Tet-On-PADI2 cells were detected using antibodies against PADI2, Beclin-1, Atg7, Atg5, Atg12, Atg5–Atg12, and β-actin at the indicated time points after 50 μM Dox exposure. **D** Representative AO staining images of Tet-On-Vector and Tet-On-PADI2 cells with 50 μM Dox treatment for 12 h, scale bar: 20 µm. The cells exhibited autophagosomes as indicated by arrowheads. **E** The bar graphs show the percentages of cells with autophagosomes with 50 μM Dox treatment for 12 h. The data are presented as the mean ± SEM of three separate experiments (***p < 0.001). The *p* value in was calculated by two-way ANOVA. **F** Following 30 µM CQ treatment, proteins extracted from Tet-On-Vector and Tet-On-PADI2 cells were detected using antibodies against LC3B, p62, cleaved caspase-3, cleaved-PARP and β-actin at the indicated time points after 50 μM Dox exposure for 24 h. **G** Proteins extracted from Tet-On-Vector and Tet-On-PADI2 cells were detected using antibodies against LAMP1, LAMP2 and β-actin at the indicated time points after 50 μM Dox exposure for 24 h. The value indicates the ratio of protein signal to β-actin signal

Autophagy is required for the removal of unfolded or aggregated proteins and the clearance of damaged organelles from the mitochondria and ER. We examined intracellular citrullinated proteins in Jurkat Tet-On cells by immunoblotting with anti-citrulline antibodies. Citrullinated proteins were found in greater abundance in Tet-On-PADI2 cells than in Tet-On-Vector cells upon Dox induction (Fig. 3A). Given that activating Jurkat T cells induces PADI2 expression and autophagy (Fig. 1), we investigated how PADI2 regulates autophagy in Jurkat T cells. PADI2 dramatically lowered SQSTM1/p62 protein levels, a well-characterized autophagy substrate, and increased LC3B-I to LC3B-II conversion, a vesicle elongation factor essential for autophagosome formation, in Tet-On-PADI2 cells following Dox stimulation. (Fig. 3B). Because overexpression of PADI2 had no effect on SQSTM1/p62 or LC3B mRNA expression (Additional file 1: Fig. S3A, B), these changes could be the result of an increase in autophagic flux induced by PADI2. As a result, we quantified the levels of the autophagy-related gene products (ATGs) Atg5 and Atg12, the Atg5–Atg12 conjugate, as well as Atg7 and Beclin-1, all of which are required for early stages of autophagy, and discovered that expression of these ATGs increased significantly in Tet-On-PADI2 cells (Fig. 3C).

AO staining of Tet-On-PADI2 cells revealed the formation of autophagosomes (Fig. 3D), and the percentage of autophagosome-containing cells was significantly higher in Tet-On-PADI2 cells than in Tet-On-Vector cells (Fig. 3E). Furthermore, chloroquine (CQ) was used to inhibit late stages of autophagy by inhibiting lysosomal acidification and protease activity, as well as to prevent autophagy by inhibiting autophagosome fusion and degradation. CQ inhibited PADI2-induced autophagy as evidenced by the accumulation of LC3B-II and p62 proteins; however, CQ did not inhibit PADI2-induced apoptosis as evidenced by an increased level of cleaved-PARP and cleaved-caspase-3 in Tet-On-PADI2 cells (Fig. 3F). Additionally, immunoblotting analysis for LAMP-1 and LAMP-2, two lysosomal markers, was performed. The levels of LAMP-1 and cleaved-LAMP-2 proteins were significantly increased in Tet-On-PADI2 cells (Fig. 3G), implying that autophagosomes were associated with an increase in lysosomes. Autophagy is widely believed to be a prosurvival process, and its dysregulation has been associated with apoptotic cell death. Our findings collectively demonstrated that overexpression of PADI2 stimulated autophagic flux in Jurkat T cells.

### PADI2 overexpression significantly increases citrullinated proteins in activated Jurkat T cells, resulting in ER stress, UPR signaling, ROS production, and ultimately autophagy

ER proteins are the predominant autophagosomal cargoes during ER stress, implying that autophagy's prosurvival effect may be due to the enhanced removal of unfolded proteins [39]. In Jurkat T cells, overexpression of PADI2 increased the level of citrullinated proteins and induced autophagy (Fig. 3A, D). Citrullinated proteins can alter the conformation of proteins and induce a severe UPR, and ER expansion can be counterbalanced by autophagy and phagocytosis during the UPR [3, 39]. As a result, we investigated whether PADI2 is involved in ER stress in this study. Autophagy activation in response to ER stress is facilitated by the PERK/eukaryotic initiation factor 2 alpha (eIF2α) pathway, which can promote autophagy gene transcription in response to stress. PERK and IRE1 are capable of inducing self-rescue or apoptotic responses via coordinated and complex signaling pathways [23]. ATF4 is a signaling molecule downstream of the PERK-eIF2α pathway, which can initiate a self-rescue response by inducing genes involved in redox reactions, stress responses, and protein secretion [40].

**Fig. 4:**
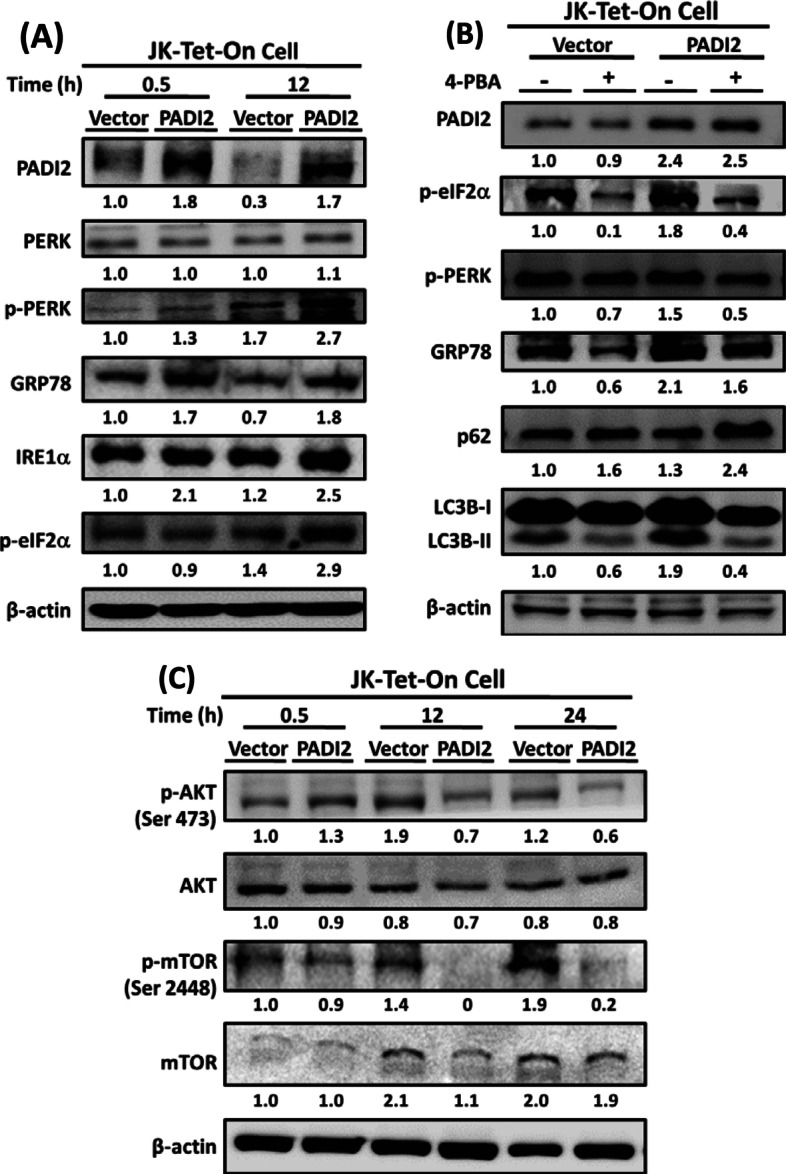
PADI2 induces ER stress-related proteins and inhibits mTOR and AKT phosphorylation in Tet-On-PADI2 Jurkat T cells. **A** Proteins extracted from Tet-On-Vector and Tet-On-PADI2 cells were detected using antibodies against PADI2, PERK, phosphorylated PERK, GRP78, IRE1α, phosphorylated eIF2α, and β-actin at the indicated time points after 50 μM Dox exposure. **B** Following 1 mM 4-PBA treatment, proteins extracted from Tet-On-Vector and Tet-On-PADI2 cells were detected using antibodies against PADI2, phosphorylated eIF2α, phosphorylated PERK, GRP78, p62, LC3B, and β-actin after 50 μM Dox exposure for 12 h. **C** Proteins extracted from Tet-On-Vector and Tet-On-PADI2 cells were detected using antibodies against AKT, phosphorylated AKT, mTOR, phosphorylated mTOR and β-actin at the indicated time points after 50 μM Dox exposure. The value indicates the ratio of protein signal to β-actin signal

Immunoblotting analysis revealed that PADI2, p-PERK, p-eIF2α, GRP78, and IRE1α protein levels were significantly increased in Tet-on-PADI2 cells (Fig. 4A). As ATF4 is a downstream signal of the PERK-eIF2α pathway, its mRNA levels were increased in Tet-On-PADI2 cells (Additional file 1: Fig. S4A). In comparison, the ER stress inhibitor 4-phenylbutyrate (4-PBA) inhibited PERK and eIF2α phosphorylation and decreased GRP78 production; additionally, 4-PBA inhibited the conversion of LC3B-I to LC3B-II and p62 degradation (Fig. 4B). 4-PBA is a small molecule that acts as a chaperone, reducing ER stress and UPR signaling [41]. Our results demonstrated that 4-PBA treatment reversed PADI2-induced ER stress and UPR signaling. Additionally, overexpression of PADI2 decreased the levels of phosphorylated AKT and mTOR in activated Jurkat T cells (Fig. 4C), while increasing intracellular ROS levels five-fold in Jurkat Tet-On-PADI2 cells compared to Tet-On-Vector cells (15.2% versus 65.7%; Additional file 1: Fig. S4B). Given the mounting evidence linking ROS production and protein unfolding, it is reasonable to conclude that citrullinated protein unfolding generates ROS as a byproduct of protein oxidation in the ER [42]. In summary, PADI2 overexpression significantly increases the amount of citrullinated proteins in activated Jurkat T cells, causing ER stress and activating UPR signaling, which induces autophagy via activation of the PERK/eIF2α/ATF4 and IRE1α/XBP1 pathway.

### Silencing of Atg12 or Atg5 inhibits PADI2-mediated autophagy and promotes PADI2-induced ER stress and apoptosis in Jurkat T cells

Atg12 and Atg5 are significant autophagic genes that regulate autophagosome elongation that contributes to the extension of the phagophoric membrane. Significant evidence suggests that inhibiting autophagy promotes cancer cell death [13, 43] and has an effect on anticancer therapies [44]. Thus, inhibiting autophagosome-lysosome fusion may impair the ability of cells to degrade unfolded proteins, predisposing them to undergo apoptosis [45]. In addition, extended ER stress reactions favor apoptosis [46].

**Fig. 5:**
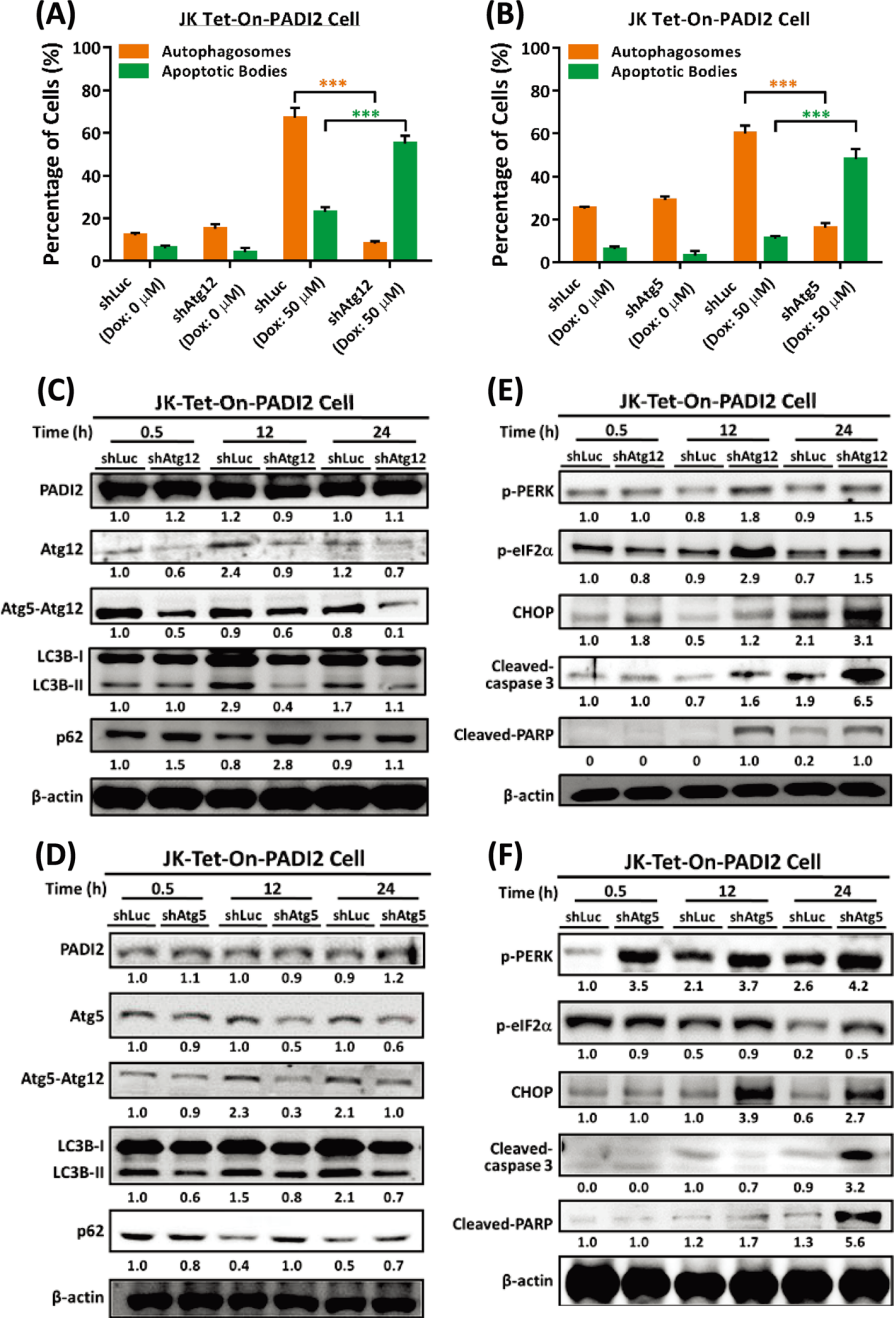
Knockdown of Atg5 or Atg12 in Tet-On-PADI2 Jurkat T cells inhibits autophagy and induces ER stress and apoptosis. The Tet-On-PADI2 Jurkat T cells with shAtg5 or shAtg12 plasmids were treated with 50 µM Dox for 12 h; “shLuc” was a scrambled RNA knockdown control. **A**, **B** The bar graphs illustrate the percentages of shAtg12- and shAtg5-Tet-On-PADI2 cells, respectively, with autophagosomes (orange bars) or apoptotic bodies (green bars) following 12 h treatment with 50 µM Dox. The data are presented as the mean ± SEM of three separate experiments (***p < 0.001). The *p* value in was calculated by one-way ANOVA. **C**, **D** Proteins extracted from shAtg12- and shAtg5-Tet-On-PADI2 cells were detected using antibodies against PADI2, Atg12, Atg5, Atg5–Atg12, LC3B, p62, and β-actin, respectively. **E**, **F** Proteins extracted from shAtg12- and shAtg5-Tet-On-PADI2 cells were detected using antibodies against p-PERK, p-eIF2α, CHOP, cleaved-caspase 3, cleaved-PARP and β-actin, respectively. The value indicates the ratio of protein signal to β-actin signal

The effect of Atg12 and Atg5 was investigated in Tet-On-PADI2 Jurkat T cell. To begin, the Atg12 or Atg5 gene was silenced in Tet-On-Vector and Tet-On-PADI2 cells (Additional file 1: Fig. S5A, B, respectively). It was evident that silencing Atg12 (shAtg12) or Atg5 (shAtg5) increased cell death in Tet-On-PADI2 cells (Additional file 1: Fig. S5C, E for Atg12, and Additional file 1: Fig. S5D, F for Atg5). AO staining of Atg12 or Atg5 knockdown in Tet-On-PADI2 cells revealed alterations in autophagosomes and apoptotic bodies (Additional file 1: Fig. S5G, H, respectively); the percentage of cells with autophagosomes was greater in shLuc-Tet-On-PADI2 cells, but the percentage of cells with apoptotic bodies was greater in shAtg12- or shAtg5-Tet-On-PADI2 cells (Fig. 5A, B, respectively), indicating that when Atg12 or Atg5 was silenced in Tet-On-PADI2 cells, PADI2-mediated autophagy was bypassed and the cell entered apoptosis.

Immunoblotting studies revealed that silencing Atg12 or Atg5 in Tet-On-PADI2 cells significantly decreased the levels of the autophagy-related proteins Atg12, the Atg5–Atg12 conjugate, and the conversion of LC3B-I to LC3B-II, as well as SQSTM1/p62 degradation (Fig. 5C, D, respectively). Due to the fact that Atg12 or Atg5 knockdown had no effect on PADI2 expression (Fig. 5C, D, respectively), these changes could be a result of a decrease in autophagic flux. Furthermore, the immunoblotting analysis revealed that knockdown of Atg12 or Atg5 in Tet-On-PADI2 cells increased significantly in the levels of the ER stress indicators p-PERK and p-eIF2α, the apoptotic proteins cleaved-PARP and cleaved-caspase 3, and the CHOP proteins, which are associated with ER stress-mediated apoptosis (Fig. 5E, F, respectively), suggesting silence of Atg12 or Atg5 in Tet-On-PADI2 cells increases PADI2-induced ER stress, which leads to apoptosis.

### Atg12 overexpression enhances PADI2-mediated autophagy and inhibits PADI2-induced ER stress and apoptosis in Jurkat T cells

**Fig. 6:**
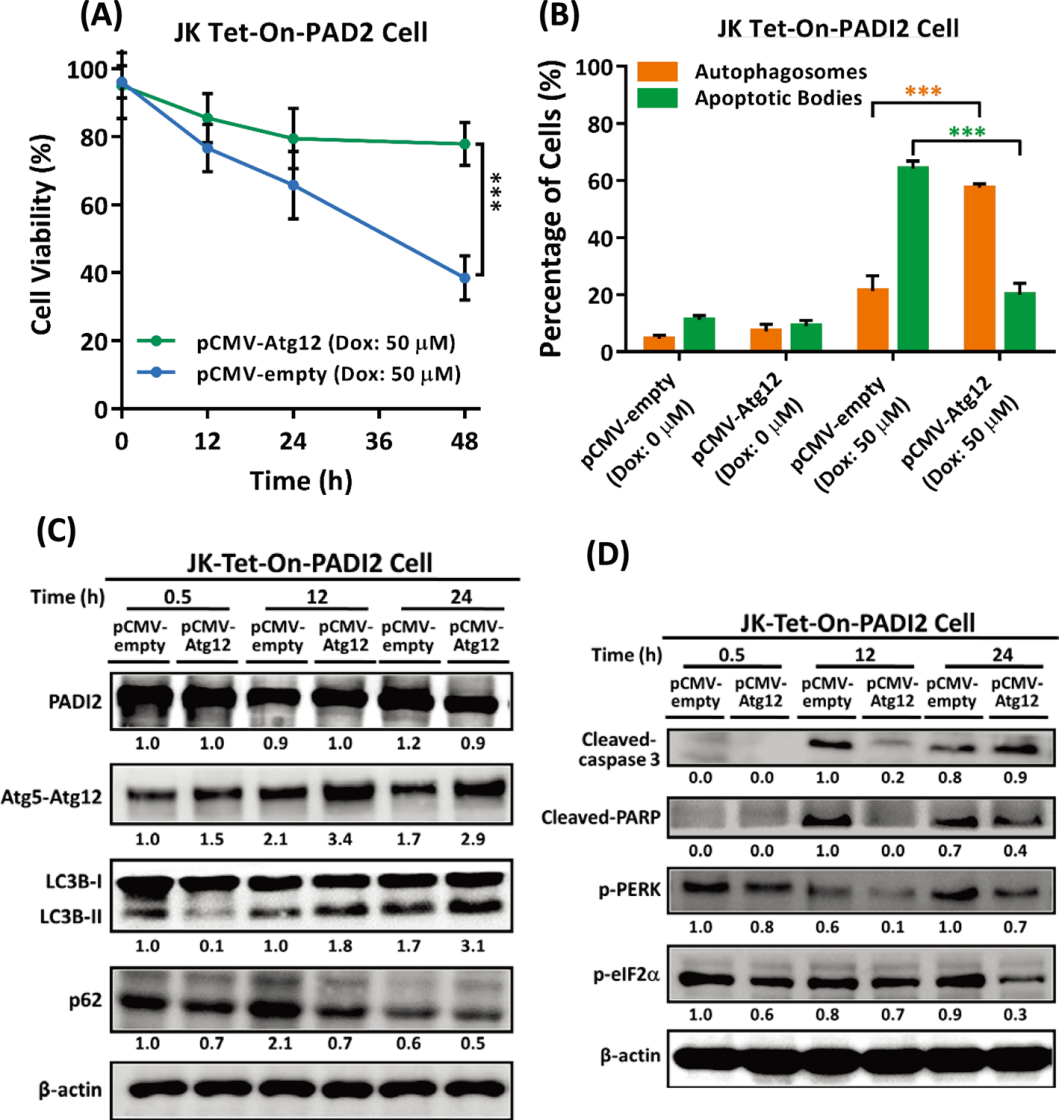
Overexpression of Atg12 in Tet-On-PADI2 Jurkat T cells enhances autophagy and inhibits ER stress and apoptosis. The Tet-On-PADI2 Jurkat T cells with pCMV-empty or pCMV-Atg12 plasmids were treated with 50 µM Dox for 24 h. **A** The viability of non-overexpressing Atg12 Tet-On-PADI2 cells and overexpressing Atg12 Tet-On-PADI2 cells in the presence of 50 µM Dox is presented as the mean ± SEM of three independent experiments (***p < 0.001). The *p* value in was determined by one-way ANOVA. **B** The bar graph illustrates the percentages of Atg12-non-overexpressing and Atg12-overexpressing Tet-On-PADI2 cells with autophagosomes (orange bars) or apoptotic bodies (green bars) following 24 h treatment with 50 µM Dox. The data are presented as the mean ± SEM of three separate experiments (***p < 0.001). The *p* value in was calculated by one-way ANOVA. **C** Proteins extracted from Atg12-non-overexpressing and Atg12-overexpressing Tet-On-PADI2 cell were detected using antibodies against PADI2, Atg5–Atg12, LC3B, p62, and β-actin. **D** Proteins extracted from Atg12-non-overexpressing and Atg12-overexpressing Tet-On-PADI2 cell were detected using antibodies against cleaved-caspase-3, cleaved-PARP, p-PERK, p-eIF2α, and β-actin, respectively. The value indicates the ratio of protein signal to β-actin signal

We constructed a pCMV-Atg12 to overexpress in Tet-On-PADI2 Jurkat T cells (Additional file 1: Fig. S6). When Tet-On-PADI2 cells were overexpressed with Atg12, cell viability was significantly increased compared to Tet-On-Vector cells (Figs. 6A); the percentage of cells with autophagosomes was greater in Atg12-overexpressing Tet-On-PADI2 cells, but the percentage of cells with apoptotic bodies was greater in Atg12-non-overexpressing Tet-On-PADI2 cells (Fig. 6B), indicating that cells survived via the autophagic pathway when the Atg12 gene was overexpressed in Tet-On-PADI2 cells. Immunoblotting analysis revealed that overexpression of Atg12 increased the levels of the Atg5–Atg12 conjugate, promoted the conversion of LC3B-I to LC3B-II, and promoted the degradation of SQSTM1/p62 (Fig. 6C); in contrast, overexpression of Atg12 decreased the levels of the apoptotic proteins cleaved-PARP and cleaved-caspase-3, as well as the ER stress-related proteins p-PERK and p-eIF2α (Fig. 6D), suggesting that Atg12 functions by reducing ER stress and apoptotic activity in the cell, thereby promoting autophagy-mediated cell survival. We concluded that silencing Atg12 (or Atg5) exacerbated ER stress and promoted apoptosis in T cells as a result of our findings. On the other hand, overexpression of Atg12 reduced ER stress and increased T cell survival in response to cell death. Taken together, these findings indicated that autophagy played a critical role in T cell survival.

### PADI2 regulates T cell activation and produces Th17 cytokines in Jurkat T cells

**Fig. 7:**
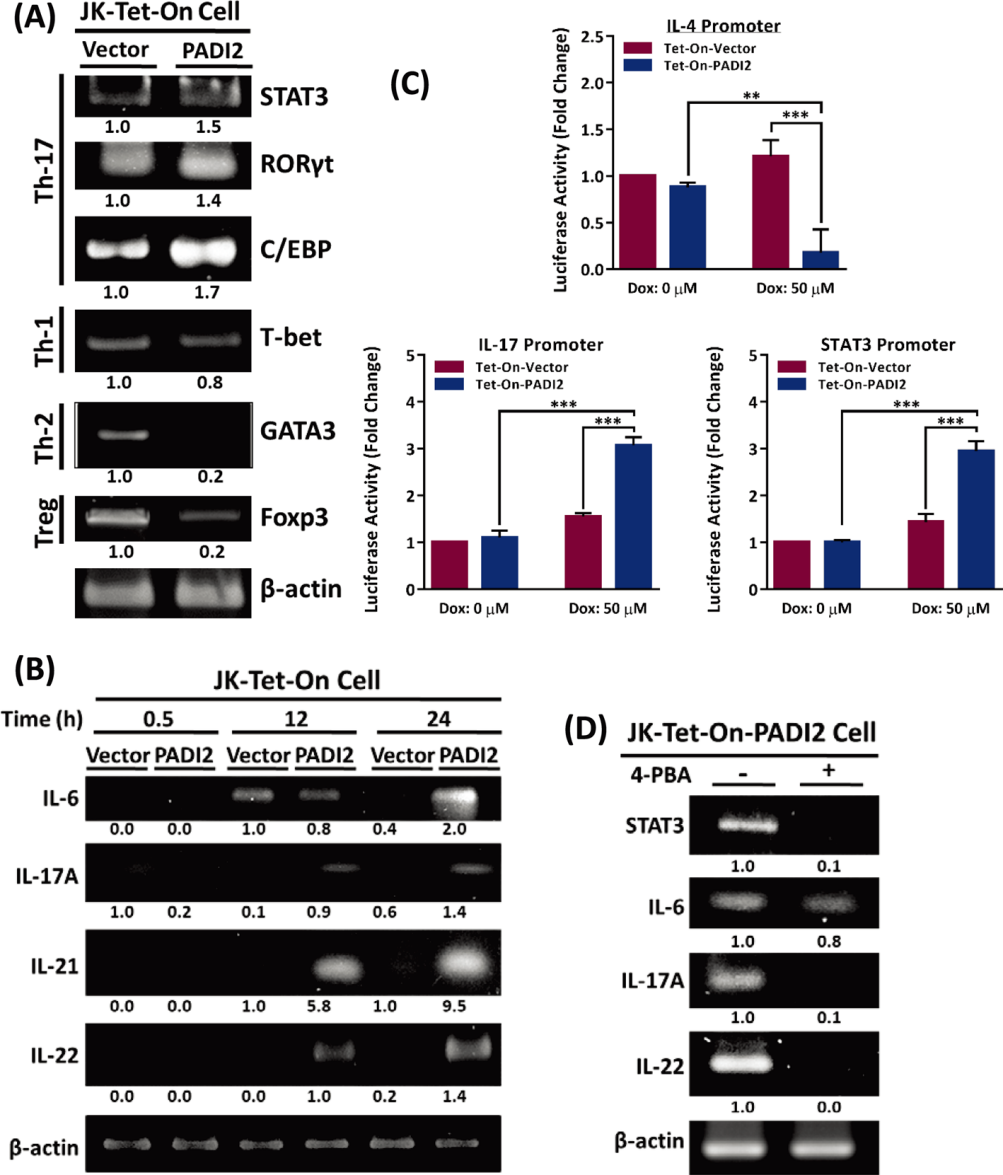
PADI2 overexpression results in the activation of T helper 17-like T cells and the production of pro-inflammatory cytokines. The Tet-On-Vector or Tet-On-PADI2 Jurkat T cells were treated with 50 µM Dox for 12 h. **A** The mRNA expression levels of the transcription factors STAT3, RORγt, C/EBP-β, T-bet, GATA3, and Foxp3 and β-actin were determined using RT-PCR. **B** RT-PCR was used to determine the levels of IL-17A, IL-22, IL-21, IL-6, and β-actin mRNA expression at 0, 12 h, and 24 h. **C** Transfection of cells with a luciferase vector containing the IL-4 promoter, the IL-17 promoter, or the STAT3 binding-site promoter was performed. After 12 h of treatment with 50 µM Dox, the luciferase activity was determined. The data are presented as the mean ± SEM of three separate experiments (**p < 0.01, and ***p < 0.001). The *p* value in was calculated by two-way ANOVA. **D** Following 4-PBA treatment, the mRNA expression levels of STAT3, IL-6, IL-17A, IL-22 and β-actin were determined. The value indicates the ratio of protein signal to β-actin signal

Inflammatory immune cells (T cells and B cells) are drawn to the target site during the immune response, resulting in the production of inflammatory cytokines, the creation of reactive oxygen species (ROS), and ER stress. It has been demonstrated that activation of Toll-like receptor (TLR) signals activates the IRE1/XBP1 pathway, resulting in the transcription of pro-inflammatory genes such as TNF-α, TGF-β, IL-2, and IL-6 [47]. As a result, the ER stress pathway is critical for the inflammatory response. We discovered that Tet-On-PADI2 Jurkat T cells had an increased mRNA level of sXBP1 (Additional file 1: Fig. S4A). Because the XBP1 protein is a transcription factor that regulates the expression of genes involved in immune system function and the cellular stress response, we investigated the relationship between PADI2 and Jurkat T cell activation and cytokine expression. PADI2 and pro-inflammatory Th17 cytokines IL-6, IL-17A, IL-17F, IL-21, and IL-22 mRNA levels increased in Jurkat Tet-On-PADI2 cells following a 12-h Dox treatment (Additional file 1: Fig. S7A). Other cytokines, such as the Th2 cytokines IL-4 and IL-13, as well as the Treg cytokine IL-10, decreased significantly; however, the Th1 cytokines IFNγ and TNF-α mRNA expression did not change significantly (Additional file 1: Fig. S7A). In Tet-On-PADI2 cells, the mRNA levels of the Th17 transcription factors STAT3, RORγt and C/EBP-β increased as well. The Th1 transcription factor T-bet mRNA expression remained constant, but the Th2 transcription factor GATA3 and the Treg transcription factor Foxp3 mRNA expression decreased (Fig. 7A). PADI2 significantly increased IL-6, IL-17A, IL-21, and IL-22 mRNA expression in Jurkat Tet-On-PADI2 cells after Dox treatment (Fig. 7B).

We investigated the effects of PADI2 on the transcriptional regulation of cytokine expression by transfecting the Tet-On-Vector and Tet-On-PADI2 cells with luciferase reporter gene constructs containing promoters for IFNγ (Th1), IL-4 (Th2), IL-17 (Th17), IL-2 (Treg), and STAT3. While Tet-On-PADI2 cells had significantly lower IL-4 promoter activity and higher IL-17 and STAT3 promoter activity than Tet-On-Vector cells (Fig. 7C); IFNγ and IL-2 promoter activity remained unchanged (Additional file 1: Fig. S7B). In Tet-On-PADI2 cells, the ER stress inhibitor 4-PBA inhibited the mRNA expression of Th17 proinflammatory cytokines IL-6, IL-17A, and IL-22, as well as STAT3 (Fig. 7D). All of these findings indicated that PADI2 promoted the activation of Th17-like T cells and the production of cytokines such as IL-6, IL-17A, IL-17F, IL-21, and IL-22 and inhibited the activation of Th2-like T cells by suppressing the production of IL-4 and IL-13.

### IL-6 silencing promotes PADI2-mediated autophagy and inhibits PADI2-induced apoptosis in Jurkat T cells

**Fig. 8:**
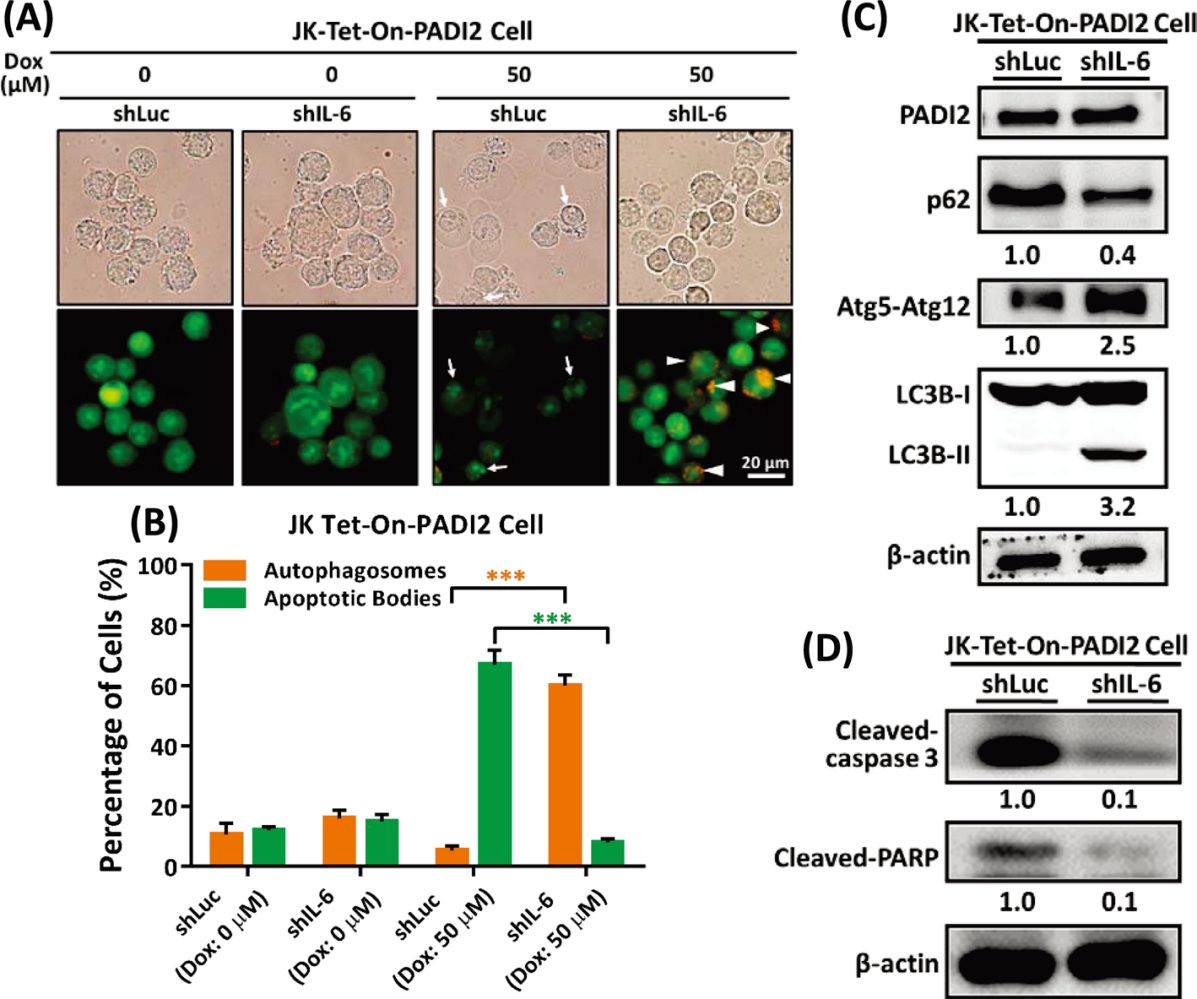
Knockdown of IL-6 in Tet-On-PADI2 Jurkat T cells promotes autophagy and inhibits apoptosis. The Tet-On-PADI2 Jurkat T cells with shIL-6 plasmids were treated with 50 µM Dox for 48 h; “shLuc” was a scrambled RNA knockdown control. **A** Representative AO staining images of shIL-6-Tet-On-PADI2 cells, scale bar: 20 µm. The cells exhibited AVOs (autophagosomes; as indicated by arrowheads). **B** The bar graph illustrates the percentages of shIL-6-Tet-On-PADI2 cells with autophagosomes (orange bars) or apoptotic bodies (green bars) following 48 h treatment with 50 µM Dox. The data are presented as the mean ± SEM of three separate experiments (***p < 0.001). The *p* value in was calculated by one-way ANOVA. **C** Proteins extracted from shIL-6-Tet-On-PADI2 cells were detected using antibodies against PADI2, p62, Atg5–Atg12, LC3B, and β-actin. **D** Proteins extracted from shIL-6-Tet-On-PADI2 cells were detected using antibodies against cleaved-caspase-3, cleaved-PARP and β-actin. The value indicates the ratio of protein signal to β-actin signal

PADI2 overexpression increases the level of the cytokine IL-6 produced by Th17 cells. (Fig. 7 and Additional file 1: Fig. S7). Numerous studies have demonstrated that overexpression of cytokines, such as IL-6, is sufficient to inhibit autophagy by promoting the Beclin-1/Bcl-2 family interaction [48]. The effect of IL-6 was further investigated in a Tet-On-PADI2 Jurkat T cell. The IL-6 gene was silenced in Tet-On-PADI2 cells (Additional file 1: Fig. S8), and AO staining revealed an increase in autophagosomes and a decrease in apoptotic bodies (Fig. 8A); the percentage of cells with autophagosomes was greater in shIL-6-Tet-On-PADI2 cells, whereas the percentage of cells with apoptotic bodies was greater in shLuc-Tet-On-PADI2 cells (Fig. 8B), indicating that when IL-6 was silenced in Tet-On-PADI2 cells, the cells entered autophagy rather than apoptosis. Immunoblotting analysis revealed that silencing IL-6 significantly increased the levels of the Atg5–Atg12 conjugate, promoted LC3B-I to LC3B-II conversion, and SQSTM1/p62 degradation in Tet-On-PADI2 cells (Fig. 8C), while silencing IL-6 also significantly decreased the levels of the apoptotic proteins cleaved-PARP and cleaved-caspase-3 (Fig. 8D), implying that IL-6 plays a function in mediating the balance of apoptosis and autophagy.

### Beclin-1 silencing increases the activation and survival of Th17-like T cells while decreasing autophagy and apoptosis

**Fig. 9:**
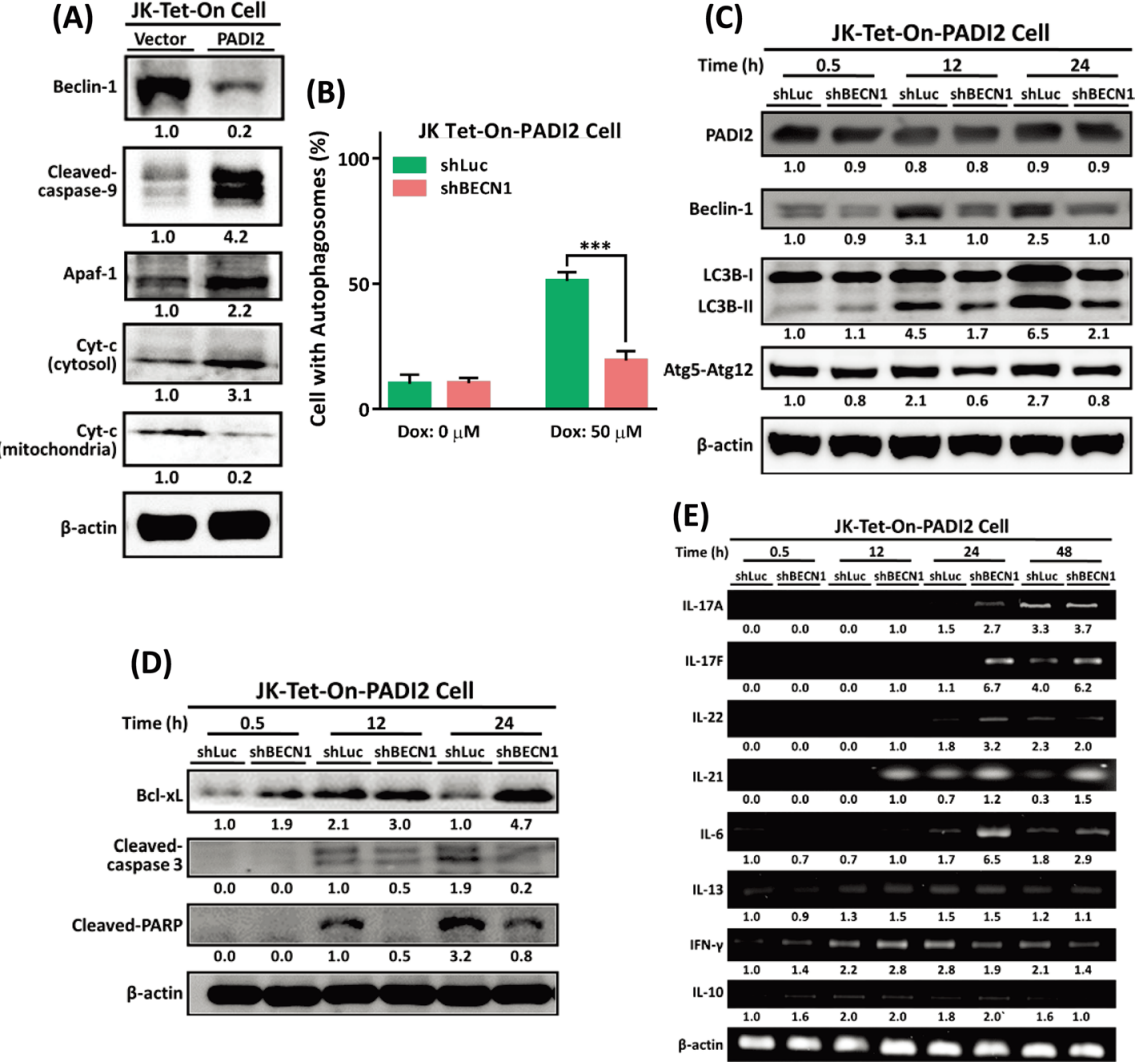
Knockdown of Beclin-1 (BECN1) in Tet-On-PADI2 Jurkat T cells inhibits autophagy, prolongs Th17-like T cell activation, and increases cell survival. The Tet-On-PADI2 Jurkat T cells with shBECN1 plasmids were treated with 50 µM Dox; “shLuc” was a scrambled RNA knockdown control. **A** Proteins extracted from Tet-On-PADI2 and Tet-On-Vector cells were detected using antibodies against Beclin-1, cleaved-caspase 9, Apaf-1, cytosolic cytochrome *c* (*Cyt-c*), mitochondrial *Cyt-c* and β-actin. The cells were treated with 50 µM Dox for 48 h. **B** The bar graph illustrates the percentages of shBECN1-Tet-On-PADI2 cells with autophagosomes. The data are presented as the mean ± SEM of three separate experiments (***p < 0.001). The *p* value in was calculated by one-way ANOVA. **C** Proteins extracted from shBECN1-Tet-On-PADI2 cells were detected using antibodies against PADI2, BECN1, LC3B, Atg5–Atg12, and β-actin. **D** Proteins extracted from shBECN1-Tet-On-PADI2 cells were detected using antibodies against Bcl-xL, cleaved-caspase 3, cleaved-PARP, and β-actin. **E** RT-PCR was used to determine the cytokine levels of IL-17A, IL-17F, IL-22, IL-21, IL-6, IL-13, IFNγ, and IL-10, and β-actin mRNA expression at 0.5 h, 12 h, 24 h, and 48 h. The value indicates the ratio of protein signal to β-actin signal

Increased expression of PADI2 stimulates the production of the Th17 cytokine IL-22 (Fig. 7 and Additional file 1: Fig. S7). It is well established that IL-22 inhibits apoptosis by activating antiapoptotic genes such as Bcl-2 and Bcl-xL. Additionally, because Bcl-2 proteins inhibit Beclin-1-dependent autophagy, IL-22 inhibits autophagy by modulating the interactions between Bcl-2/Bcl-xL and Beclin-1 [49]. Caspase-mediated cleavage of Beclin-1 inhibits the autophagy-inducing activity of Beclin-1 and generates pro-apoptotic Beclin-1 fragments that induce mitochondrial cytochrome c release. As a result, we discovered that in the late stage (48 h), overexpression of PADI2 increased Beclin-1 protein degradation and apoptosome formation, which is composed of apoptotic protease activating factor 1 (Apaf-1), cytochrome c, and caspase-9 (Fig. 9A).

The effect of Beclin-1 was further investigated in Tet-On-PADI2 Jurkat T cell. The Beclin-1 gene was silenced in Tet-On-PADI2 cells (Additional file 1: Fig. S9A), and silencing Beclin-1 slightly increased cell viability in Tet-On-PADI2 cells (Additional file 1: Fig. S9B). The AO staining data indicated that silencing beclin-1 resulted in a significant reduction in autophagosome formation in Tet-On-PADI2 cells (Additional file 1: Fig. S9C); the percentage of cells with autophagosomes was lower in shBeclin-1-Tet-On-PADI2 cells than in shLuc-Tet-On-PADI2 cells (Fig. 9B), indicating that when Beclin-1 was silenced in Tet-On-PADI2 cells, the cells did not enter PADI2-induced autophagy. Therefore, the immunoblotting analysis revealed that silencing Beclin-1 in Tet-On-PADI2 cells decreased the levels of the Atg5–Atg12 conjugate significantly and inhibited LC3B-I to LC3B-II conversion (Fig. 9C).

The Bcl-2 family acts as a dual regulator, preventing both autophagy and apoptosis. Caspase-mediated cleavage of Beclin-1 activated by death-induced signaling impairs autophagic signals and promotes apoptosis. Our results reveal that silencing Beclin-1 increases Bcl-xL expression and decreases cleaved-PARP and cleaved-caspase-3 levels, indicating that silencing Beclin-1 may protect T cells from PADI2-induced apoptosis (Fig. 9D). Furthermore, the Bcl-2 protein family is required for T cell activation. Our results also reveal that silencing Beclin-1 increases Th17-like T cell activation, as evidenced by the early-stage mRNA expression levels of the Th17 cytokines IL-17A, IL-17F, IL-21, IL-22, and IL-6 (Fig. 9E). These findings suggested that silencing Beclin-1 enhance the activation and survival of Th17-like T cells while attenuating autophagy and apoptosis.

### PADI2 silencing alleviates ER stress caused by PADI2 and decreases Th17-like T cell activation-related cytokine expression and ACAD

**Fig. 10:**
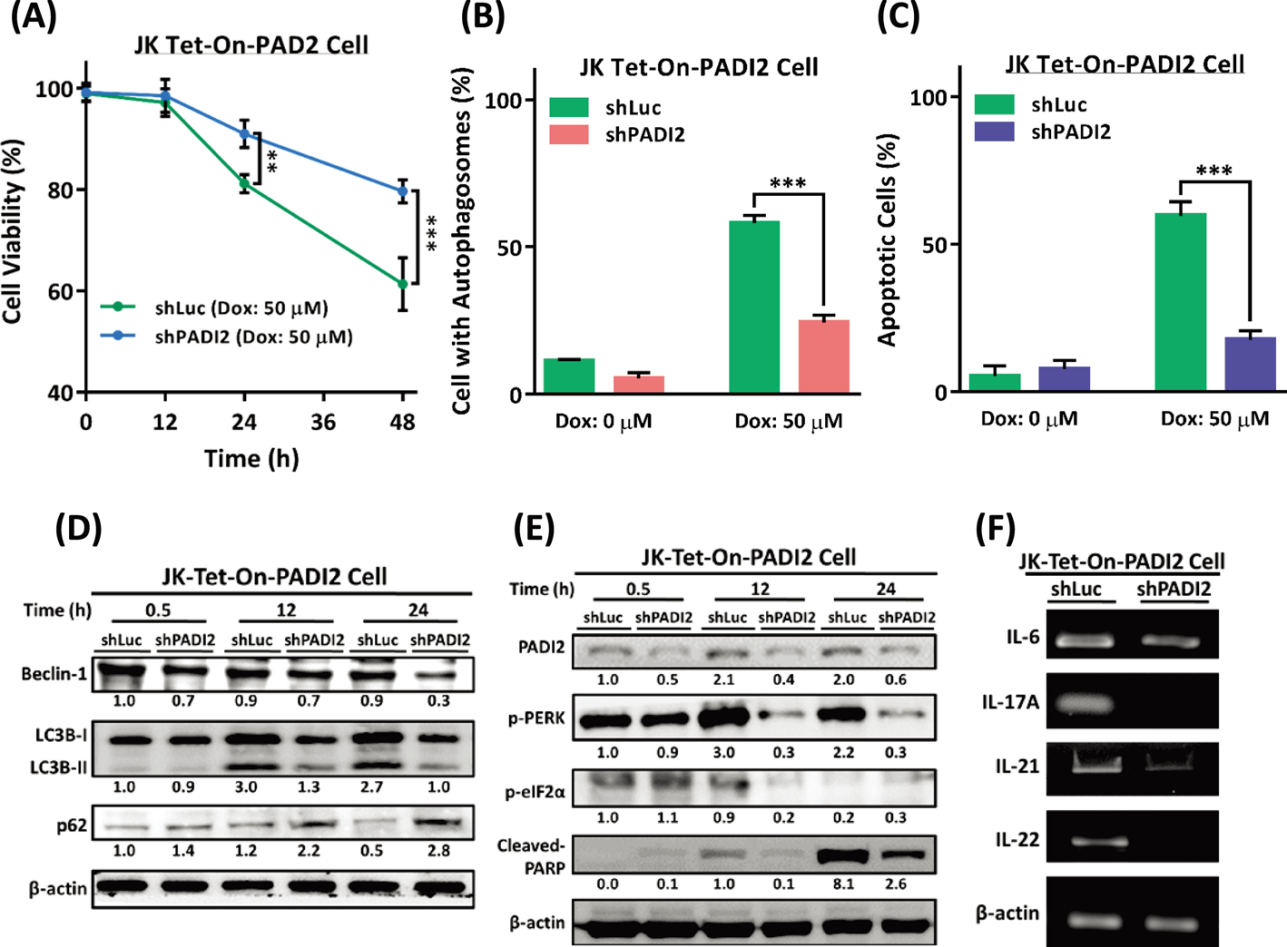
PADI2 silencing has the opposite effect on PADI2-induced ER stress, autophagy, and Th17-like T cell activation in Tet-On-PADI2 Jurkat T cells. The Tet-On-PADI2 Jurkat T cells with shLuc or shPADI2 plasmids were treated with 50 µM Dox for 24 h or 48 h; “shLuc” was a scrambled RNA knockdown control. **A** The viability of shLuc- and shPADI2-Tet-On-PADI2 cells with 50 µM Dox is presented as the mean ± SEM of three independent experiments (**p < 0.01, and ***p < 0.001). The *p* value in was determined by one-way ANOVA. **B**, **C** The bar graphs depict the percentages of shLuc- and shPADI2-Tet-On-PADI2 cells with autophagosomes (orange bars) or apoptotic bodies (green bars) after 24 or 48 h of 50 µM Dox treatment, respectively. The data are presented as the mean ± SEM of three separate experiments (***p < 0.001). The *p* value in was calculated by one-way ANOVA. **D** Proteins extracted from shLuc- and shPADI2-Tet-On-PADI2 cells were detected using antibodies against BECN1, LC3B, p62, and β-actin. **E** Proteins extracted from shLuc- and shPADI2-Tet-On-PADI2 cells were detected using antibodies against PADI2, p-PERK, p-eIF2α, cleaved-PARP, and β-actin. **F** RT-PCR was used to determine the levels of IL-6, IL-17A, IL-21, IL-22, and β-actin mRNA expression. The value indicates the ratio of protein signal to β-actin signal

We finally examined the effects of PADI2 silencing (Additional file 1: Fig. S10), finding that the cells survived when PADI2 was silenced in Tet-On-PADI2 cells (Fig. 10A), and both the percentage of cells with autophagosomes and apoptotic bodies were significantly lower in shPADI2-Tet-On-PADI2 cells than in shLuc-Tet-On-PADI2 cells (Fig. 10B, C, respectively).

Immunoblotting studies revealed that silencing PADI2 significantly decreased the levels of Beclin-1, the conversion of LC3B-I to LC3B-II, as well as SQSTM1/p62 degradation (Fig. 10D). When PADI2 was silenced, the ER stress indicators p-PERK and p-eIF2α, the apoptotic protein cleaved-PARP, and the mRNA levels of the Th17 cytokines IL-6, IL-17A, IL-21, and IL-22 all decreased significantly (Fig. 10E, F). In summary, these findings indicate that inhibiting PADI2 alleviates PADI2-mediated ER stress while simultaneously decreasing Th17-like T cell activation-related cytokine expression and ACAD.

### PADI2 induces ER stress and autophagy by producing citrullinated proteins and regulating cytokine expression

TPA, a diacylglycerol-related phorbol ester, directly binds to and activates PKC [50]. Ca^2+^ influx is stimulated by the calcium ionophore Ion [51]. Thus, TPA plus Ion mimics the signal elicited by antigens that initiate lymphocyte activation [52]. In this study, we discovered that treating Jurkat T cells with TPA and Ion increased T cell activation and PADI2 expression (Fig. 1). PADI2 is expressed in synovial tissue by inflammatory cells such as lymphocytes and monocytes. Citrullinated proteins are found in inflammatory cells. The PADI2 promoter contains four Sp1 binding sites, implying that TPA is involved in its regulation [53].

**Fig. 11:**
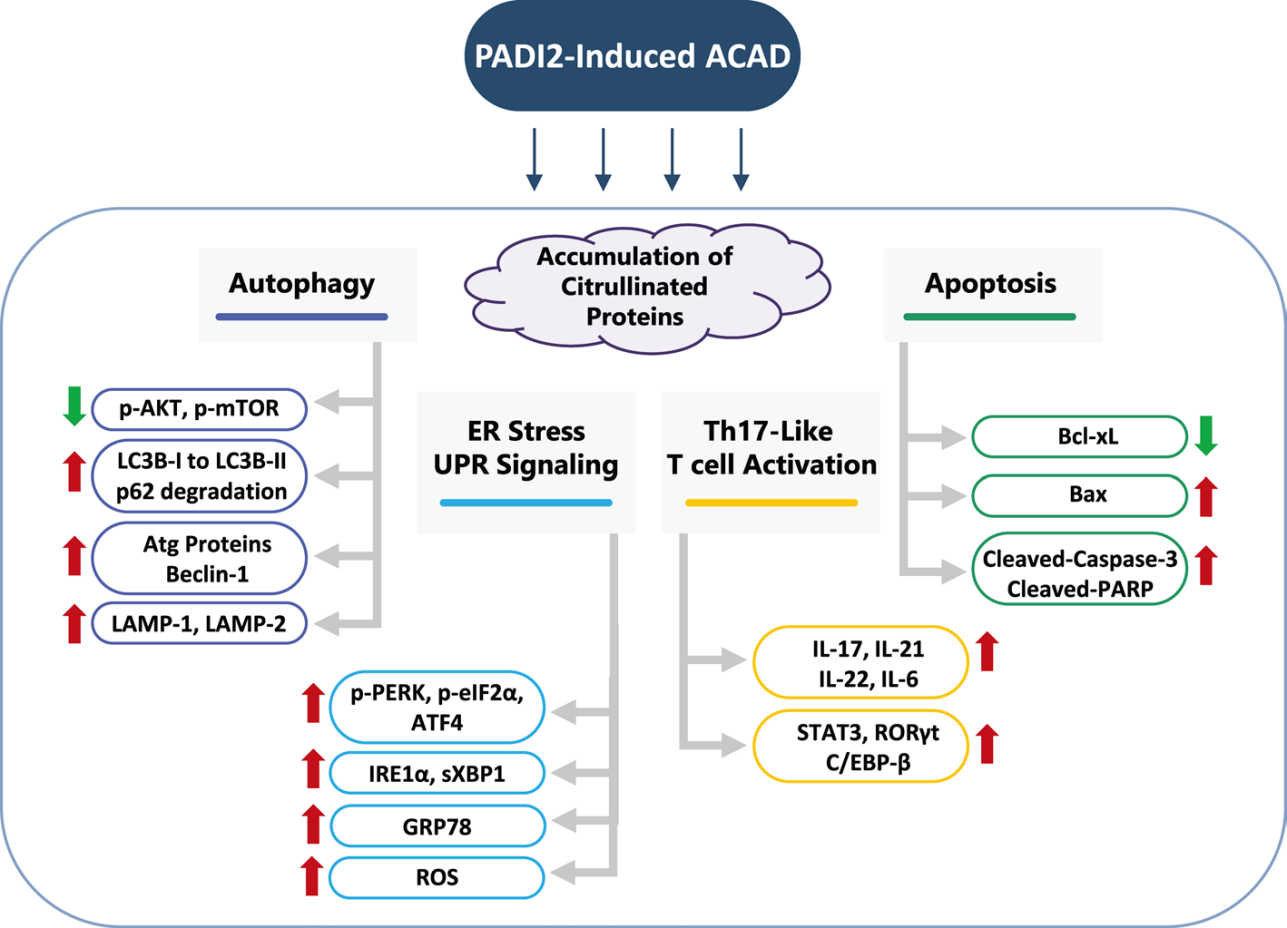
Simplified model illustrating how the overexpression of PADI2 could induce endoplasmic reticulum (ER) stress and autophagy. When PADI2 is activated or overexpressed, citrullination and accumulation of cellular proteins occur, resulting in ER stress and induces UPR signaling via activation of the PERK/eIF2α pathway, as well as the production of ROS to induce autophagy. Additionally, PADI2 stimulates the activation of Th17-like T cells and the production of pro-inflammatory cytokines such as IL-6, IL-17, IL-21, and IL-22, ultimately resulting in the ACAD

Increased levels of PADI2 results in protein citrullination and unfolding (Fig. 3A). Increased accumulation of citrullinated proteins in the ER results in ER stress and activation of the UPR, which involves the induction of molecular chaperones and ER-associated degradation to prevent cell death (Fig. 11). ER stress has been associated with autophagy, which plays a critical role in the ER stress response’s survival and death decision. ER stress promotes autophagy’s survival, possibly by augmenting the removal of unfolded proteins. Our studies demonstrated that PADI2 induced increases in p-PERK, p-eIF2α, GRP78, IRE1α, sXBP1, and ATF4 via the UPR pathway (Fig. 11). eIF2α phosphorylation enables the translation of ATF4, a transcription factor that regulates the transcription of autophagy-related genes such as Atg12. The ER transports unfolded proteins to autophagosomes and lysosomes [16], which degrade proteins and eliminate cellular stress [24].

Autophagy and the activity of the Bcl-2 family are two crucial mechanisms in the selection of cell fate. Anti-apoptotic Bcl-2 proteins have been shown to interact with the autophagy regulator Beclin-1, including Bcl-2 [49], Bcl-xL [54], Bcl-w [55], and MCL-1 [56]. Structural and mutational studies show that Beclin-1 contains a functional BH3 motif that mediates binding to anti-apoptotic proteins [57]. The structure of the Beclin-1 BH3 motif, which is inserted into the hydrophobic groove on Bcl-xL in a manner similar to that of other BH3 domains on pro-apoptotic proteins, supports this finding [58]. Numerous studies indicate that caspases cleave Beclin-1 at specific sites during apoptosis, inhibiting cyto-protective autophagy in cells that have committed to apoptotic cell death. Depending on the intensity and duration of stress, a combination of Bcl-2-dependent regulation and feedback loops between Beclin-1 and caspases enforces sequential activation of cellular responses. Our findings showed that knocking down Beclin-1 increased Bcl-xL expression and decreased cleavage of apoptotic proteins (cleaved-caspase-3 and cleaved PARP), resulting in the cell escaped from apoptosis (Fig. 9). Because activation of PADI2 increased Beclin-1 expression, the anti-apoptotic function of Bcl-xL was suppressed, and the cell entered apoptosis (Fig. 11).

ACAD is widely believed to be associated with T lymphocyte cytokine withdrawal-induced apoptosis (CWIA). PADI2 induced the production of Th17 cytokines and resulted in the death of activated T cells (Fig. 7). PADI2 may contribute to autoimmunity in Jurkat T cells by producing citrullinated proteins and regulating cytokine expression. PADI2 promoted the production of Th17-like cytokines such as IL-17A, IL-17F, IL-21, IL-22, and IL-6 (Fig. 11). Th17 cells, first described in mice, are the primary source of IL-17 in a variety of types of adaptive immunity. Th17 cells are critical in initiating inflammation [59] and in initiating the immediate protective response to foreign pathogens. Additionally, Th17 cells are involved in autoimmune diseases such as rheumatoid arthritis, multiple sclerosis, psoriasis, and lupus.

## Conclusion

Numerous autoimmune diseases have been associated with citrullinated proteins that have been identified as autoantigens in RA and other autoimmune diseases. Citrullinated proteins are generated as a result of PADI-mediated posttranslational modification. We demonstrated that PADI2 promoted Th17 cytokines via both ER stress and the autophagy coupling pathway, ultimately resulting in Jurkat T cells undergoing ACAD. PADI2 may exert control over Th17 activation and ACAD via ER stress regulation and autophagy regulation. These findings suggest that in the future, inhibiting Th17 T cell activation and the development of severe autoimmune diseases may be possible by specifically targeting PADI2 with novel antagonists.

## Supplementary Information

**Additional file 1: Figure S1.** TPA and Ion induce autophagy and apoptosis in T-ALL cells, as represented by AO staining images. **Figure S2.** Overexpression of PADI2 in Jurkat T cells. **Figure S3.** LC3B or p62 mRNA expression in Tet-On-PADI2 Jurkat T cells. **Figure S4.** ATF4, uXBP1 and sXBP1 mRNA expression and ROS production in Tet-On-PADI2 Jurkat T cells. **Figure S5.** Atg12 and Atg5 protein levels in shAtg12- and shAtg5-Tet-On-PADI2 cells, as well as cell viability. **Figure S6.** Atg12 protein levels in Tet-On-PADI2 Jurkat T cells, as well as cell viability. **Figure S7.** PADI2 overexpression results in the activation of T helper 17-like T cells and the production of pro-inflammatory cytokines. **Figure S8.** IL-6 protein level in shIL-6-Tet-On-PADI2 cells. **Figure S9.** BECN1 protein levels in Tet-On-PADI2 Jurkat T cells, as well as cell viability. **Figure S10.** PADI2 protein levels in Tet-On-PADI2 Jurkat T cells, as well as cell viability. **Table S1.** Primer sequences used to amplify the following target genes.

## Data Availability

The data that supports the findings of this study are available in Additional file of this article.

## Abbreviations

4-PBA: 4-Phenylbutyrate
ACAD: Activated T cell-autonomous death
Apaf-1: Apoptotic protease activating factor 1
ATF4 and 6: Activating transcription factor 4 and 6
Atg: Autophagy-related gene
Bax: Bcl-2-associated X protein
Bcl-2: B-cell lymphoma 2
CHOP: C/EBP homologous protein
CQ: Chloroquine
DCFH-DA: Dichloro-dihydro-fluorescein diacetate
DMSO: Dimethyl sulfoxide
Dox: Doxycycline
EGFP: Enhanced green fluorescent protein
eIF2: Eukaryotic initiation factor 2α
ER: Endoplasmic reticulum
GRP78: Glucose-regulated protein 78
IRE1α: Inositol-requiring enzyme 1α
LAMP: Lysosome-associated membrane protein
MAP1LC3B: Microtubule-associated protein 1 light chain 3B
PADI: Peptidylarginine deiminase
PARP: Poly (ADP-ribose) polymerase
PERK: PRKR-like endoplasmic reticulum kinase
RA: Rheumatoid arthritis
ROS: Reactive oxygen species
SQSTM1: Sequestosome 1
XBP1: X-box binding protein-1
TLR: Toll-like receptors
TPA: 12-*O*-Tetradecanoylphorbol-13-acetate
UPR: Unfolded protein response
