# Supplementary material for: Peptidylarginine deiminase 2 promotes T helper 17-like T cell activation and activated T cell-autonomous death (ACAD) through an endoplasmic reticulum stress and autophagy coupling mechanism
Source: Cell Mol Biol Lett. 2022 Mar 2;27:19. doi: 10.1186/s11658-022-00312-0 (PMC8903576; doi:10.1186/s11658-022-00312-0)
Supplement: Supplementary file 1 — Additional file 1: Figure S1. TPA and Ion induce autophagy and apoptosis in T-ALL cells, as represented by AO staining images. Figure S2. Overexpression of PADI2 in Jurkat T cells. Figure S3. LC3B or p62 mRNA expression in Tet-On-PADI2 Jurkat T cells. Figure S4. ATF4, uXBP1 and sXBP1 mRNA expression and ROS production in Tet-On-PADI2 Jurkat T cells. Figure S5. Atg12 and Atg5 protein levels in shAtg12- and shAtg5-Tet-On-PADI2 cells, as well as cell viability. Figure S6. Atg12 protein levels in Tet-On-PADI2 Jurkat T cells, as well as cell viability. Figure S7. PADI2 overexpression results in the activation of T helper 17-like T cells and the production of pro-inflammatory cytokines. Figure S8. IL-6 protein level in shIL-6-Tet-On-PADI2 cells. Figure S9. BECN1 protein levels in Tet-On-PADI2 Jurkat T cells, as well as cell viability. Figure S10. PADI2 protein levels in Tet-On-PADI2 Jurkat T cells, as well as cell viability. Table S1. Primer sequences used to amplify the following target genes. [file 11658_2022_312_MOESM1_ESM.docx]

**Additional file 1**

**Peptidylarginine Deiminase 2 Promotes T Helper 17-Like T Cell Activation and Activated T Cell-Autonomous Death (ACAD) through an Endoplasmic Reticulum Stress and Autophagy Coupling Mechanism**

Yi-Fang Yang, Chuang-Ming Wang, I-Hsin Hsiao, Yi-Liang Liu, Wen-Hao Lin, Chih-Li Lin, Hui-Chih Hung and Guang-Yaw Liu

**
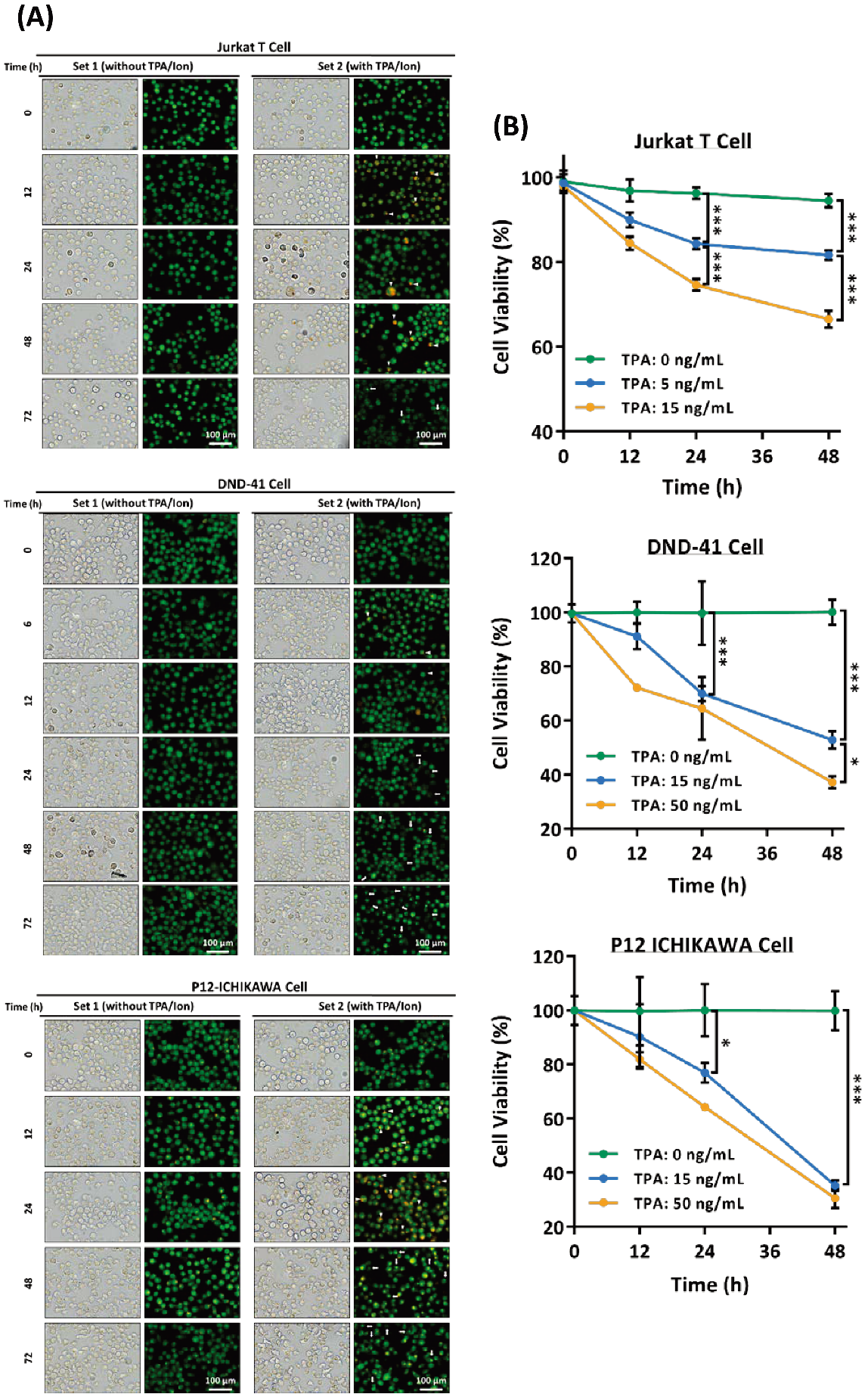
**

**Figure S1. TPA and Ion induce autophagy and apoptosis in T-ALL cells, as represented by AO staining images. (A)** Representative AO staining images of Jurkat T, DND-41, and P12-ICHIKAWA cells, scale bar: 100 µm. The cells exhibited acidic vesicular organelles (AVOs) (autophagosomes, orange; as indicated by arrowheads), or apoptotic bodies (green; as indicated by arrows). **(B)** Cell viability of Jurkat T, DND-41, and P12-ICHIKAWA cells with Ion and different doses of TPA (0-50 ng/mL) treatment was determined by trypan blue exclusion. The concentration of Ion for Jurkat T, DND-41, and P12-ICHIKAWA cells was fixed at 1, 3, and 3 μM, respectively. The data are presented as the mean ± SEM of three independent experiments (**p*<0.05, and ****p*<0.001). The *p* value in was determined by one-way ANOVA.

**
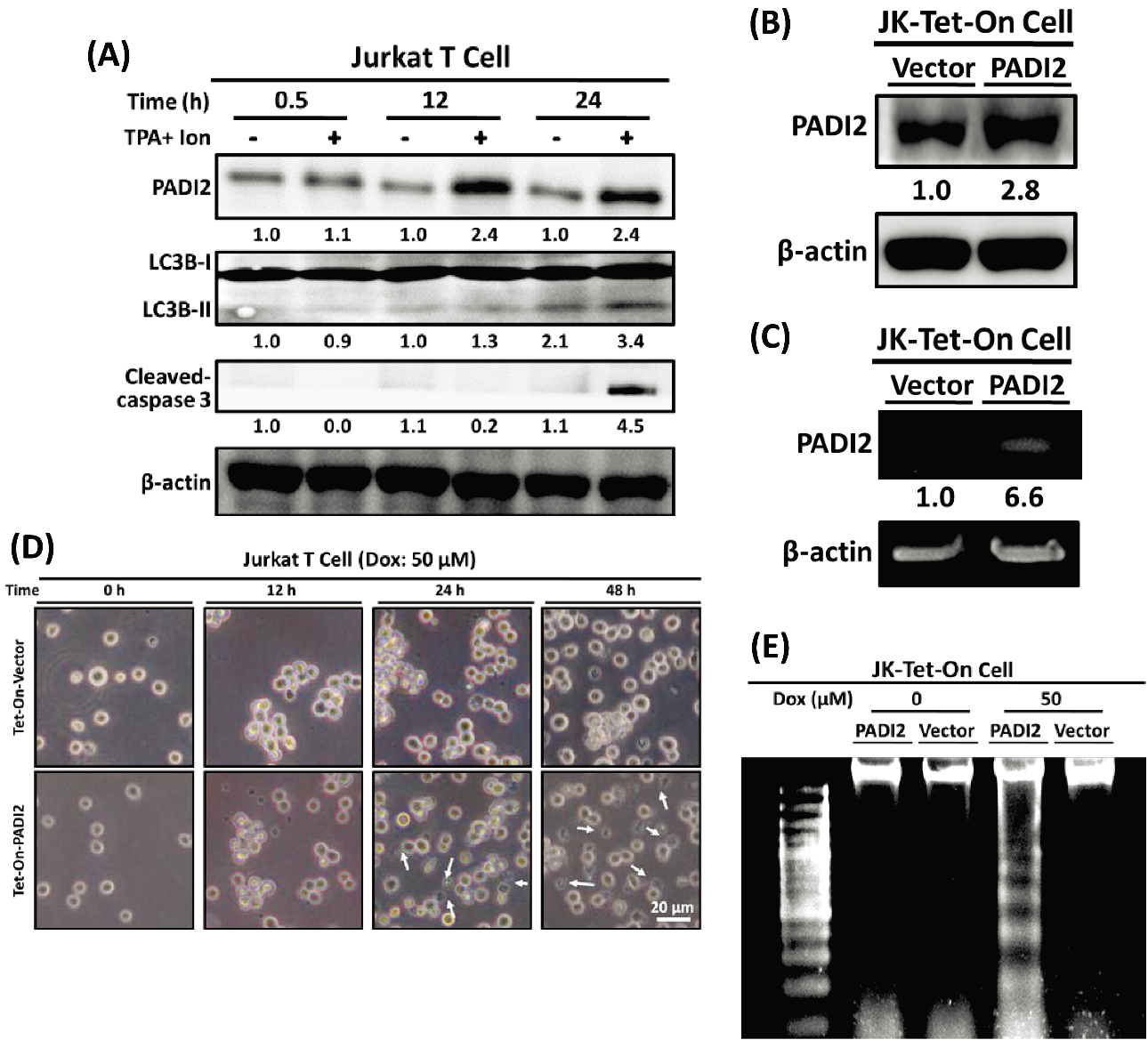
**

**Figure S2. Overexpression of PADI2 in Jurkat T cells. (A)** Time-dependent immunoblotting demonstrated the protein levels of PADI2, LC3B, and cleaved-caspase-3 in Jurkat T cells in the absence or presence of TPA (15 μg/mL) and Ion (1 μM) as the indicated time. **(B)** Antibodies against PADI2 and β-actin were used to detect proteins extracted from Tet-On-Vector and Tet-On-PADI2 cells 12 hours after 50 μM Dox exposure. **(C)** Reverse transcription-polymerase chain reaction (RT-PCR) analysis was used to detect PADI2 mRNA. **(D)** Representative light micrographs of Tet-On-Vector and Tet-On-PADI2 cells, scale bar: 20 µm. The cells developed apoptotic bodies after being exposed to 50 μM Dox for 0 h, 12 h, 24 h, and 48 h, as indicated by the arrows. Quantitative data was depicted on Figure 2B. **(E)** A 2% DNA agarose gel was used to determine DNA fragmentation in Tet-On-PADI2 and Tet-On-Vector cells following 48 h treatment with 50 μM Dox. The value indicates the ratio of protein signal to β-actin signal.

**
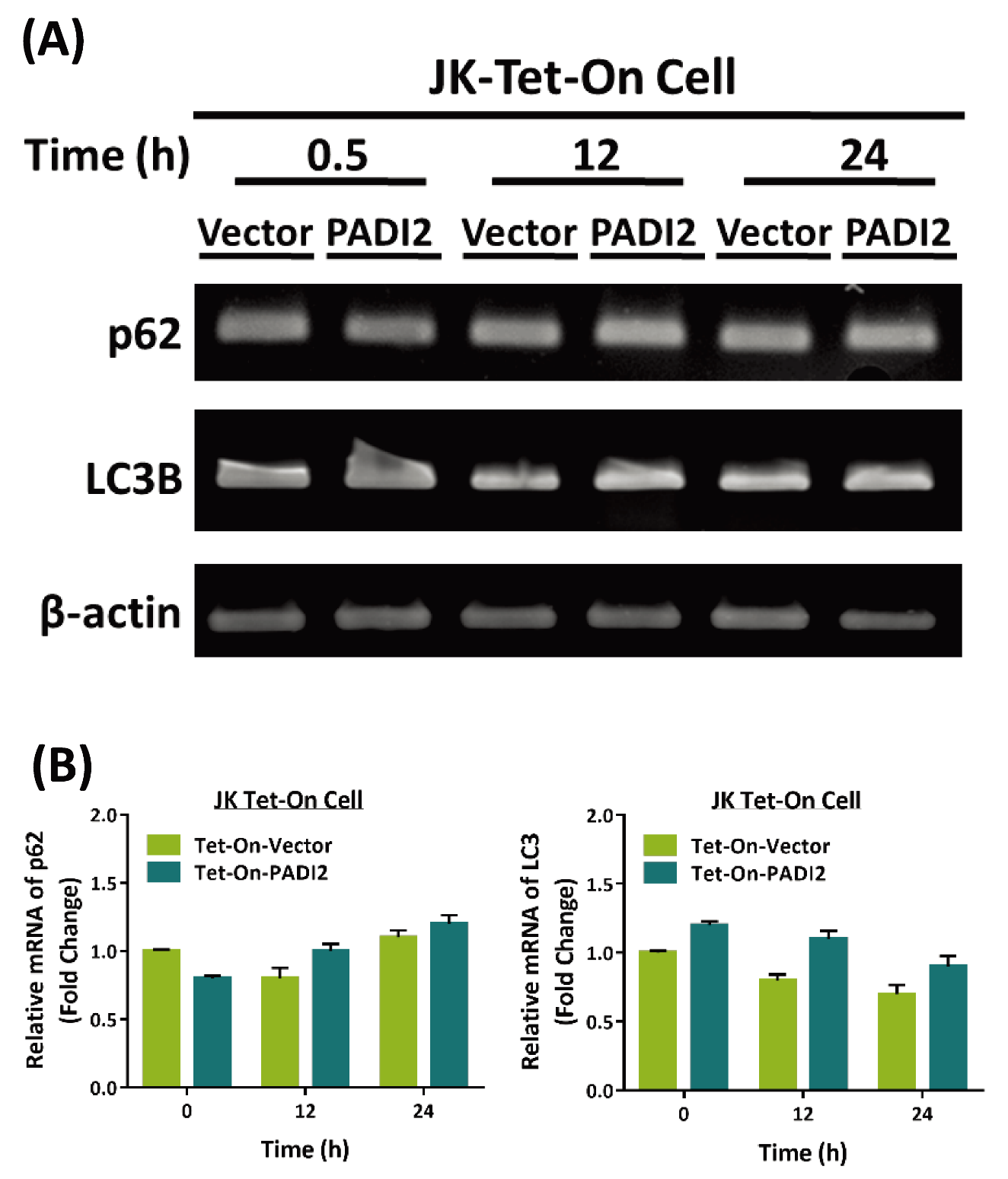
**

**Figure S3. LC3B or p62 mRNA expression in Tet-On-PADI2 Jurkat T cells. (A)** The mRNA expression levels of p62 and LC3B in Tet-On-Vector and Tet-On-PADI2 cells upon 50 μM Dox treatment were detected by reverse transcription-polymerase chain reaction (RT-PCR). **(B)** The bar graphs show the relative mRNA level of p62 and LC3B in Tet-On-Vector cells and Tet-On-PADI2 cells upon 50 μM Dox treatment. The data are presented as the mean ± SEM of three separate experiments.

**
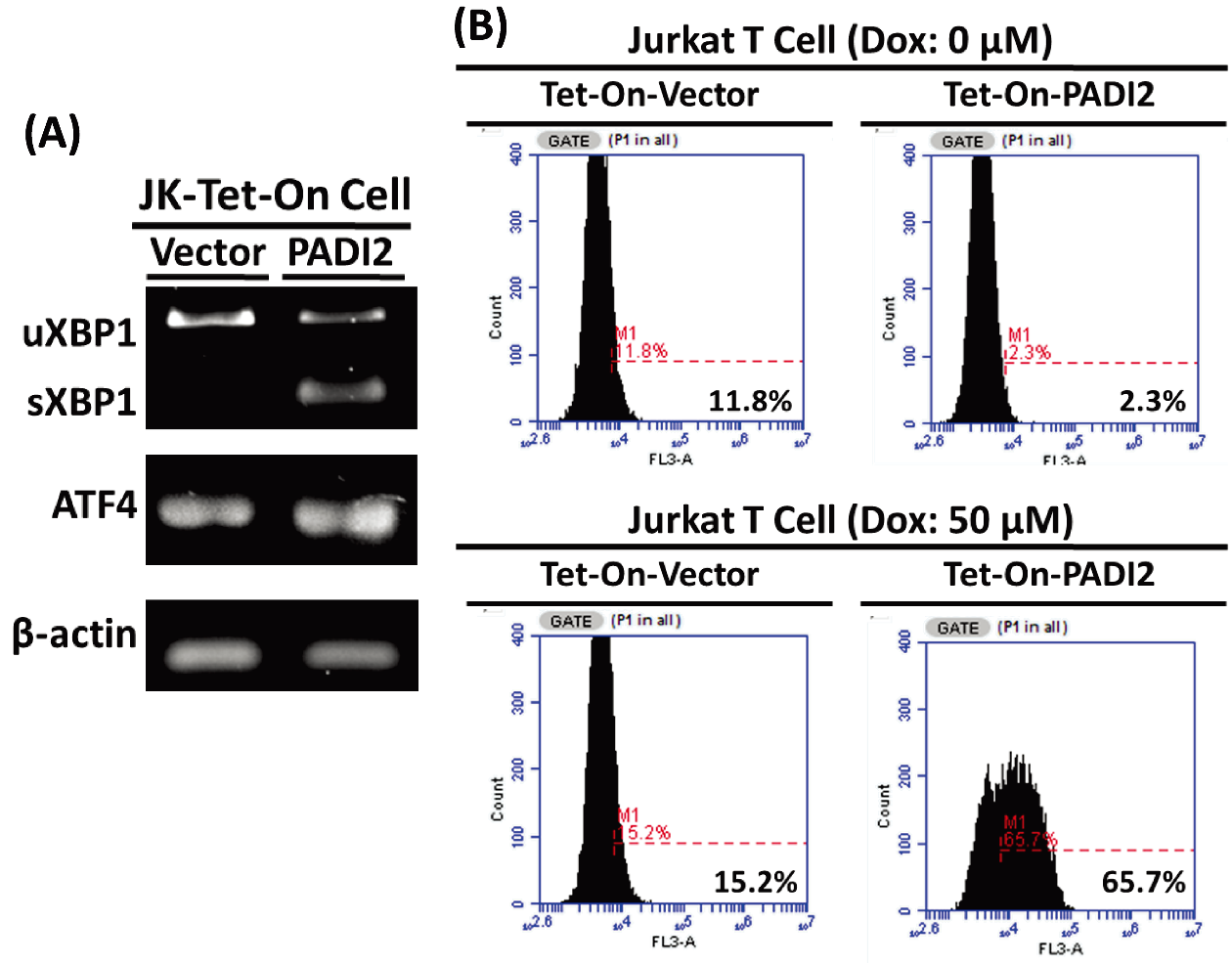
**

**Figure S4. ATF4, uXBP1 and sXBP1 mRNA expression and ROS production in Tet-On-PADI2 Jurkat T cells. (A)** The mRNA expression levels of uXBP1, sXBP1 and ATF4 in Tet-On-Vector and Tet-On-PADI2 cells upon 50 μM Dox treatment were detected by RT-PCR. **(B)** The production of reactive oxygen species (ROS) was determined using flow cytometry with 2'-7'-dichlorodihydrofluorescein diacetate (DCFH-DA).

**
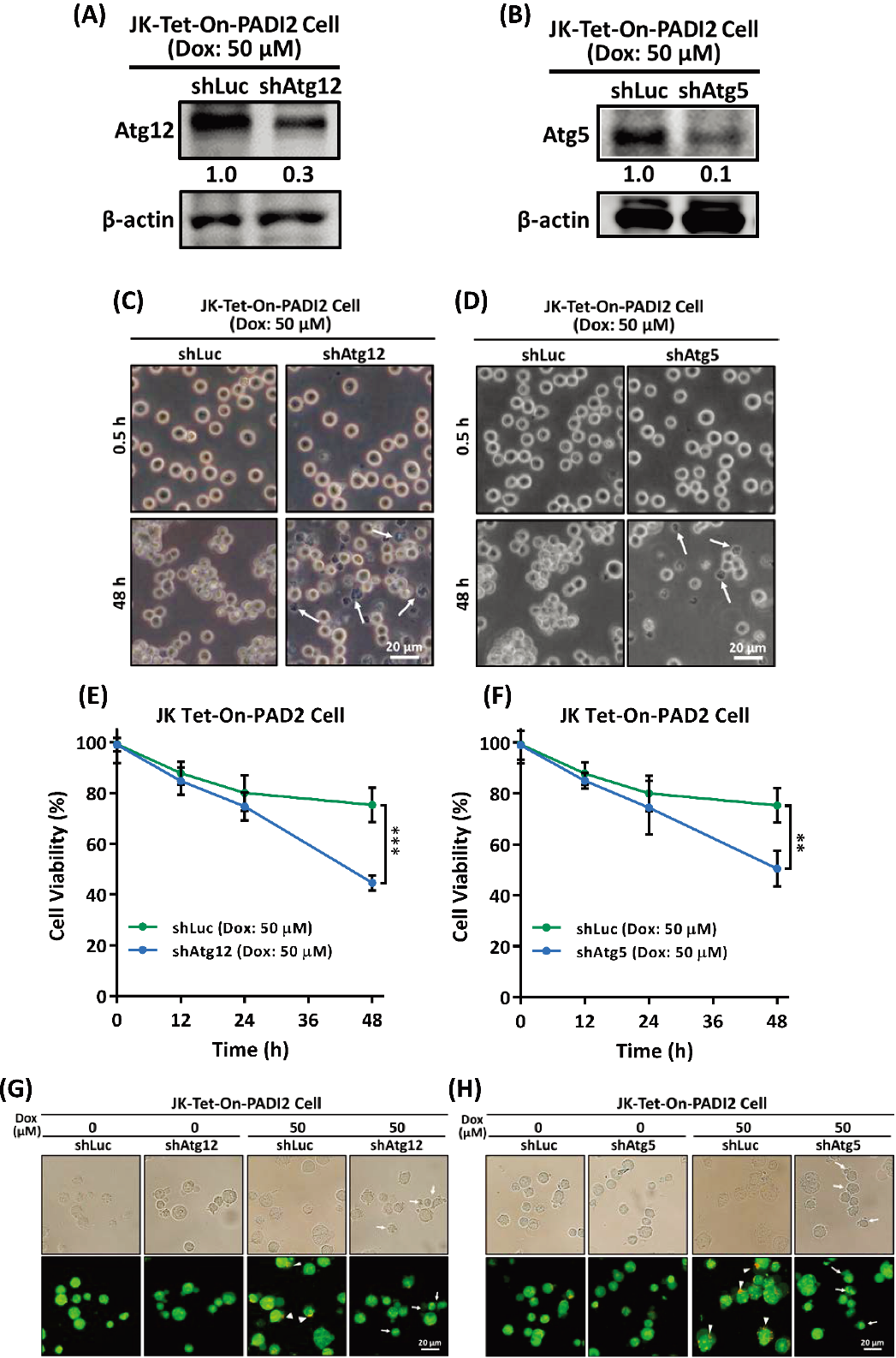
**

**Figure S5. Atg12 and Atg5 protein levels in shAtg12- and shAtg5-Tet-On-PADI2 cells, as well as cell viability**

**(A)** and **(B)** Antibodies against Atg12, Atg5, and β-actin were used to determine the protein levels of Atg12 and Atg5. The value indicates the ratio of protein signal to β-actin signal. **(C)** and **(D)** At the indicated times, the morphologies of shAtg12- and shAtg5-Tet-On-PADI2 cells were observed using a light microscope, respectively. **(E) and (F)** Cell viability of shAtg12- and shAtg5-Tet-On-PADI2 cells, respectively, was determined by trypan blue exclusion. The data are presented as the mean ± SEM of three independent experiments (***p*<0.01, and ****p*<0.001). The *p* value in was determined by one-way ANOVA. **(G) and (H)** Representative AO staining images of shAtg12- and shAtg5-Tet-On-PADI2 cells, respectively, scale bar: 20 µm. The cells exhibited AVOs (autophagosomes; as indicated by arrowheads), or apoptotic bodies (as indicated by arrows). Quantitative data was depicted on Figures 5A and 5B, respectively.

**
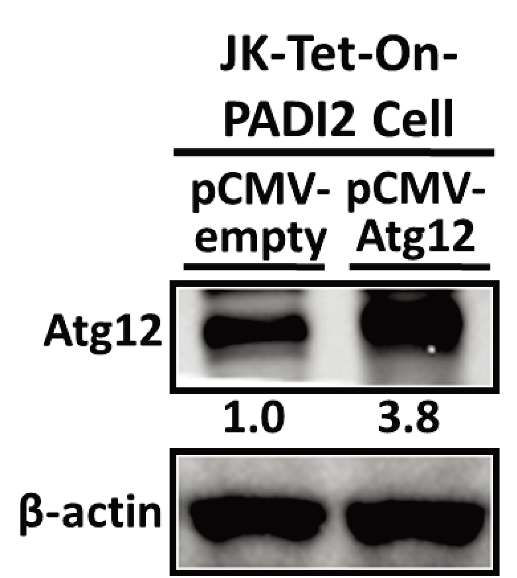
**

**Figure S6. Atg12 protein levels in Tet-On-PADI2** **Jurkat T cells, as well as cell viability**

Antibodies against Atg12 and β-actin were used to determine the protein levels of Atg12 in Atg12-non-overexpressing and Atg12-overexpressing Tet-On-PADI2 cells. The value indicates the ratio of protein signal to β-actin signal.

**
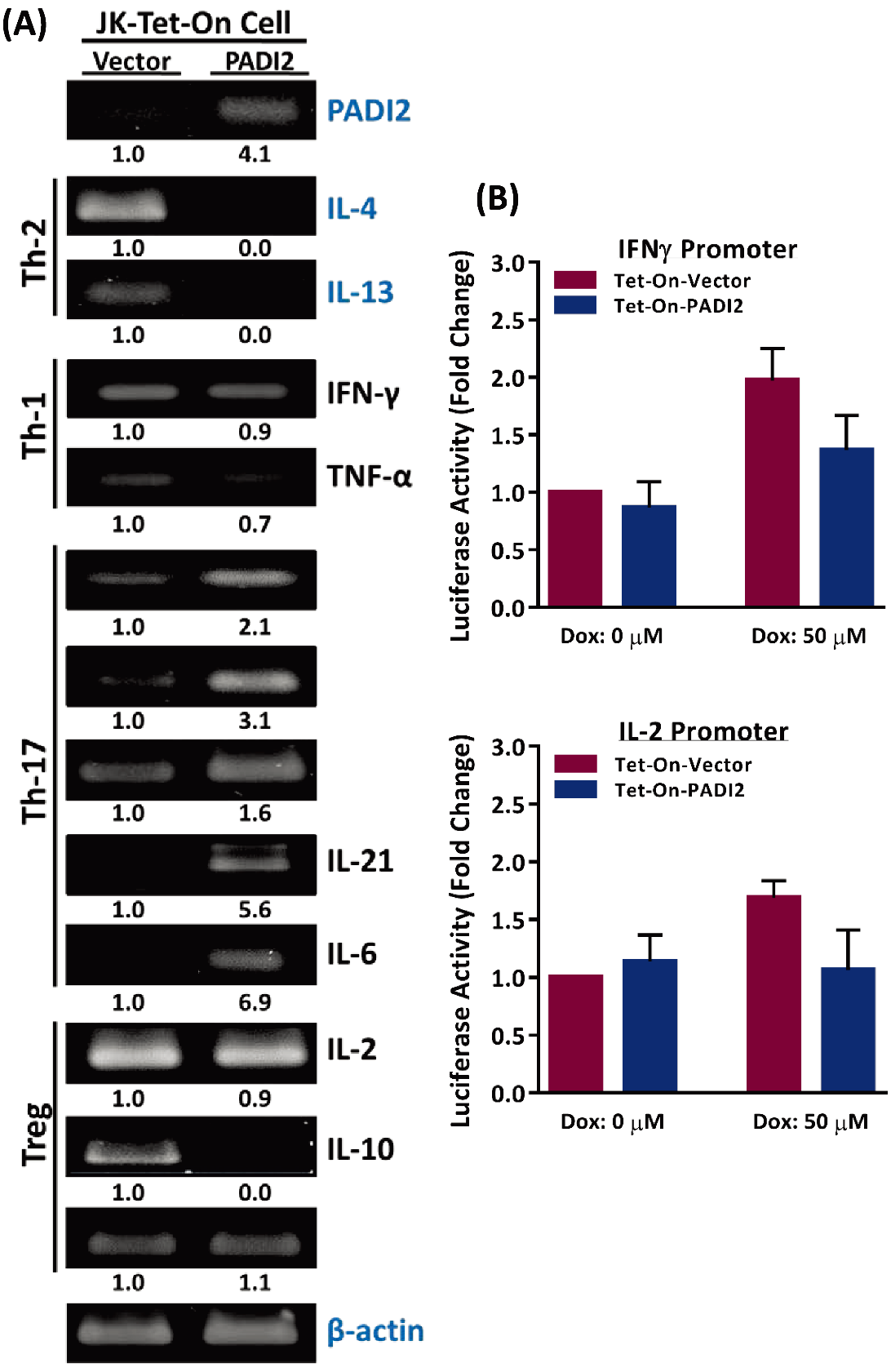
**

**Figure S7. PADI2 overexpression results in the activation of T helper 17-like T cells and the production of pro-inflammatory cytokines**

The Tet-On-Vector or Tet-On-PADI2 Jurkat T cells were treated with 50 µM Dox for 12 h. **(A)** The mRNA expression levels of PADI2, IL-4, IL-13, IFNγ, TNFα, IL-6, IL-17A, IL-17F, IL-21, IL-22, IL-2, IL-10, TGFβ and β-actin were determined using RT-PCR. The data for PADI2, IL-4, IL-13, IL-17A, IL-17F and β-actin (in blue) were published in our previous study (Chang et al., 2016). **(B)** Transfection of cells with a luciferase vector containing an IFNγ or IL-2 promoter. The luciferase activity was determined after treatment with 50 µM Dox for 12 hours. The value is the ratio of the mRNA's signal for protein to that for β-actin.

**
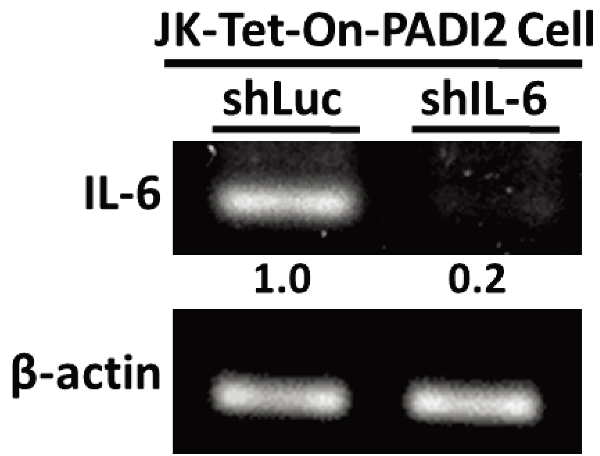
**

**Figure S8. IL-6 protein level in shIL-6-Tet-On-PADI2 cells**

RT-PCR was used to determine the levels of IL-6 and β-actin mRNA expression. The value is the ratio of the mRNA's signal for protein to that for β-actin.

**
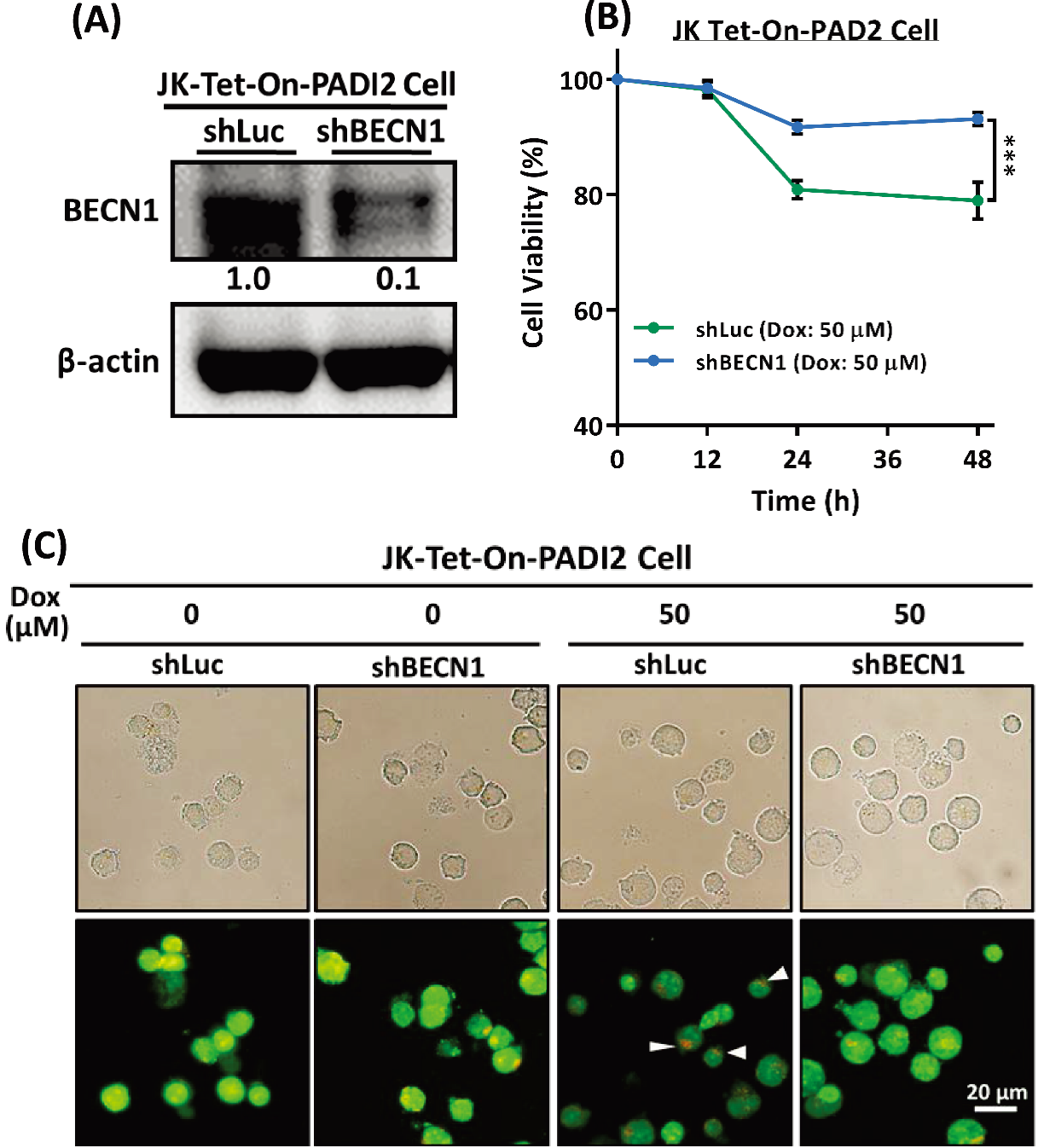
**

**Figure S9. BECN1 protein levels in Tet-On-PADI2 Jurkat T cells, as well as cell viability**

**(A)** Antibodies against BECN1 and β-actin were used to determine the protein levels of BECN1 in Tet-On-PADI2 cells. The number represents the ratio of protein/β-actin bands' signal. **(B)** The viability of shBECN1-Tet-On-PADI2 cells in the presence of 50 µM Dox is presented as the mean ± SEM of three independent experiments (****p*<0.001). The *p* value in was determined by one-way ANOVA. **(C)** Representative AO staining images of shBECN1-Tet-On-PADI2 cells, scale bar: 20 µm. The cells exhibited AVOs (autophagosomes; as indicated by arrowheads). Quantitative data was depicted on Figure 9B.

**
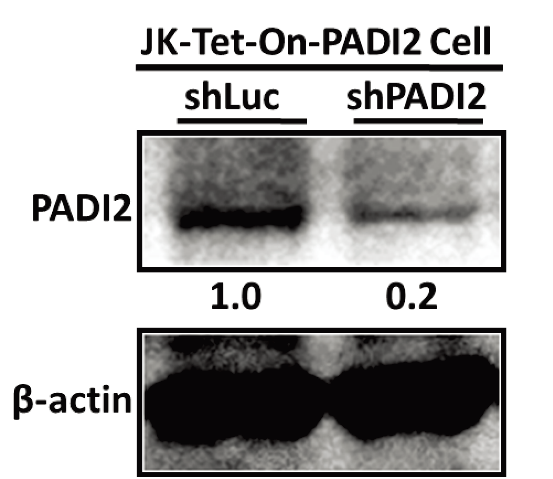
**

**Figure S10. PADI2 protein levels in Tet-On-PADI2 Jurkat T cells, as well as cell viability**

Antibodies against PADI2 and β-actin were used to determine the protein levels of PADI2 in Tet-On-PADI2 cells. The number represents the ratio of protein/β-actin bands' signal.

| Table S1: Primer sequences used to amplify the following target genes | | |
| --- | --- | --- |
|  | Forward primer | Reverse primer |
| β-actin | 5’-AGCGGGAAATCGTGCGTG-3’ | CAGGGTACATGGTGGTGC |
| ATF4 | GTTCTCCAGCGACAAGGCTA | ATCCTCCTTGCTGTTGTTGG |
| C/EBP-β | ACAGCGACGAGTACAAGATCC | GCAGCTGCTTGAACAAGTTCC |
| FOXP3 | TCATCCGCTGGGCCATCCTG | GTGGAAACCTCACTTCTTGGTC |
| GATA3 | TCATTAAGCCCAAGCGAAGG | GTCCCCATTGGCATTCCTC |
| IFNγ | GGATCCATGCAGGACCCA | GAATTCTTACTGGGATGCTCTTC |
| IL-10 | TCAAGGCGCATGTGAACTCC | GATGTCAAACTCACTCATGGCT |
| IL-13 | GCAATGGCAGCATGGTATGG | AAGGAATTTTACCCCTCCCTAACC |
| IL-17A | AGATTACTACAACCGATCCACCT | GGGGACAGAGTTCATGTGGTA |
| IL-17F | GCTGTCGATATTGGGGCTTG | GGAAACGCGCTGGTTTTCAT |
| IL-2 | AACTCACCAGGATGCTCACATTTA | TTCCTGGGTCTTAAGTGAAAGTTT |
| IL-21 | GCAGGGAGAAGACAGAAACA | GGAATCTTCACTTCCGTGTG |
| IL-22 | TTCTCTTGGCCCTCTTGGTA | TTCTCCCCAATGAGACGAAC |
| IL-4 | CCAACTGCTTCCCCCTCTG | TCTGTTACGGTCAACTCGGTG |
| IL-6 | TAGCCGCCCCACACAGACAG | GGCTGGCATTTGTGGTTGGG |
| LC3B | CGGAGAAGACCTTCAAGCAG | CTGGGAGGCATAGACCATGT |
| p62 | AAGCCGGGTGGGAATGTTG | GCTTGGCCCTTCGGATTCT |
| PADI2 | ACCTCTGGACCGATGTCTACA | TCCCTTCCTCGTCATAGTAGTTG |
| RORγt | CTGCTGAGAAGGACAGGGAG | AGTTCTGCTGACGGGTGC |
| STAT3 | GGAGGAGTTGCAGCAAAAAG | TGTGTTTGTGCCCAGAATGT |
| sXBP1 | CTGAGTCCGAATCAGGTGCAG | ATCCATGGGGAGATGTTCTGG |
| T-bet | GATGTTTGTGGACGTGGTCTTG | CTTTCCACACTGCACCCACTT |
| TGF-β | CAACAATTCCTGGCGATACC | GAACCCGTTGATGTCCACTT |
| TNF-α | GTGACAAGCCTGTAGCCCA | ACTCGGCAAAGTCGAGATAG |
